# Characterisation of genetic regulatory effects for osteoporosis risk variants in human osteoclasts

**DOI:** 10.1186/s13059-020-01997-2

**Published:** 2020-03-26

**Authors:** Benjamin H. Mullin, Jennifer Tickner, Kun Zhu, Jacob Kenny, Shelby Mullin, Suzanne J. Brown, Frank Dudbridge, Nathan J. Pavlos, Edward S. Mocarski, John P. Walsh, Jiake Xu, Scott G. Wilson

**Affiliations:** 1 Department of Endocrinology & Diabetes, Sir Charles Gairdner Hospital, Nedlands, WA Australia; 2 School of Biomedical Sciences, The University of Western Australia, Crawley, WA 6009 Australia; 3 Department of Health Sciences, University of Leicester, Leicester, UK; 4 Department of Microbiology and Immunology, Emory Vaccine Center, School of Medicine, Emory University, Atlanta, GA USA; 5 Medical School, The University of Western Australia, Crawley, WA Australia; 6 Department of Twin Research & Genetic Epidemiology, King’s College London, London, UK

**Keywords:** Osteoclast, Osteoporosis, GWAS, eQTL, BMD, Fracture, RIPK3, RIP3, FBN2, SNP

## Abstract

**Background:**

Osteoporosis is a complex disease with a strong genetic contribution. A recently published genome-wide association study (GWAS) for estimated bone mineral density (eBMD) identified 1103 independent genome-wide significant association signals. Most of these variants are non-coding, suggesting that regulatory effects may drive many of the associations. To identify genes with a role in osteoporosis, we integrate the eBMD GWAS association results with those from our previous osteoclast expression quantitative trait locus (eQTL) dataset.

**Results:**

We identify sixty-nine significant *cis*-eQTL effects for eBMD GWAS variants after correction for multiple testing. We detect co-localisation of eBMD GWAS and osteoclast eQTL association signals for 21 of the 69 loci, implicating a number of genes including *CCR5*, *ZBTB38*, *CPE*, *GNA12*, *RIPK3*, *IQGAP1* and *FLCN*. Summary-data-based Mendelian Randomisation analysis of the eBMD GWAS and osteoclast eQTL datasets identifies significant associations for 53 genes, with *TULP4* presenting as a strong candidate for pleiotropic effects on eBMD and gene expression in osteoclasts. By performing analysis using the GARFIELD software, we demonstrate significant enrichment of osteoporosis risk variants among high-confidence osteoclast eQTL across multiple GWAS *P* value thresholds. Mice lacking one of the genes of interest, the apoptosis/necroptosis gene *RIPK3*, show disturbed bone micro-architecture and increased osteoclast number, highlighting a new biological pathway relevant to osteoporosis.

**Conclusion:**

We utilise a unique osteoclast eQTL dataset to identify a number of potential effector genes for osteoporosis risk variants, which will help focus functional studies in this area.

## Background

Genome-wide association studies (GWAS) have successfully identified thousands of genetic variants associated with complex traits and diseases in humans. However, the vast majority of these variants lie in non-coding regions of the genome such as intronic or intergenic DNA, and it is thought that many of them influence the phenotype through regulatory effects on nearby genes. For many of these variants, the identity of the effector gene responsible for the association remains to be determined. This is further complicated by the fact that the closest gene to the GWAS association signal is not always the effector gene, and in some cases, neither is the most plausible biological candidate gene from the region. Expression quantitative trait locus (eQTL) studies in disease-relevant cell/tissue types provide valuable information on this issue by characterising associations between genetic variants and expression of nearby genes. The Genotype-Tissue Expression (GTEx) project provides extensive eQTL data for many tissues [1]; however, not all cell types are represented in that resource and many eQTL studies have used complex tissues containing a mixture of cell types. Apart from an eQTL study performed on human osteoblasts cultured from surgical explants of bone [2], there has historically been a lack of eQTL studies performed in cell types relevant to osteoporosis. We recently generated a unique osteoclast-specific eQTL resource using cells obtained from 158 subjects to fill the void of eQTL data for that cell type and for the study of human bone disease [3, 4].

Bone is a dynamic tissue that is constantly being remodelled through the coupled actions of osteoblasts and osteoclasts. Osteoporosis is the most common metabolic bone disease in humans and is characterised by reduced bone mineral density (BMD), micro-architectural deterioration of the bone tissue and an increased risk of fracture. Excess mortality caused by osteoporotic fracture has been estimated at 9% in women and 24% in men at 1 year postfracture, and 24% in women and 26% in men at 5 years postfracture [5]. Postmenopausal women are at particularly high risk of developing osteoporosis due to reduced oestrogen levels. Certain environmental factors such as dietary calcium intake and exercise have also been shown to influence an individual’s risk of developing the disease [6, 7]. In addition to these factors, osteoporosis has a strong heritable component, with twin and family studies generating heritability estimates of 0.46–0.84 for BMD [8–10]. Individuals with an affected first-degree relative have an elevated estimated familial relative risk for fragility fracture of 1.31–4.24 [11, 12].

GWAS have proven to be very successful at identifying genetic variants associated with bone parameters [13–18]. A recently published GWAS for estimated BMD (eBMD), a trait calculated using quantitative ultrasound measurements at the heel, included 426,824 individuals from the UK Biobank and is the largest GWAS ever performed for a bone density phenotype [19]. The study identified 1103 conditionally independent genome-wide significant association signals located in 518 loci, of which 301 are novel loci [19]. Like most complex disease GWAS, the majority of these signals are led by non-coding variants. In order to identify putative effector genes and genetic regulatory mechanisms with a role in osteoporosis, particularly those that are relevant to bone resorbing cells, we integrated the results from that study with our osteoclast eQTL dataset and bone phenotypes from knockout mice.

## Results

Population characteristics of the 158 women recruited into the osteoclast eQTL study cohort are presented in Additional file 1: Table S1. All participants had self-reported European ancestry, and principal components analysis failed to identify any ethnic outliers. After genotype imputation and QC, genotype data was available for 5,373,348 variants in the study sample with a minor allele frequency (MAF) ≥ 5% and an IMPUTE2 info score ≥ 0.4. After applying QC criteria to the gene expression dataset, a total of 15,688 expressed gene transcripts were identified in the osteoclast-like cells.

### Identification of eQTL associations for eBMD GWAS variants

Of the 1103 lead genetic variants recently identified as independently associated with eBMD at the genome-wide significance level, 929 were present in the osteoclast eQTL dataset (MAF ≥ 5% in the cohort and had an expressed gene located within the ± 1 Mb analysis window). The vast majority of those missing were low frequency variants with a MAF < 5% in the cohort. For the 929 variants present in the dataset, a mean of 15.0 genes was present within the ± 1 Mb analysis window.

**Fig. 1:**
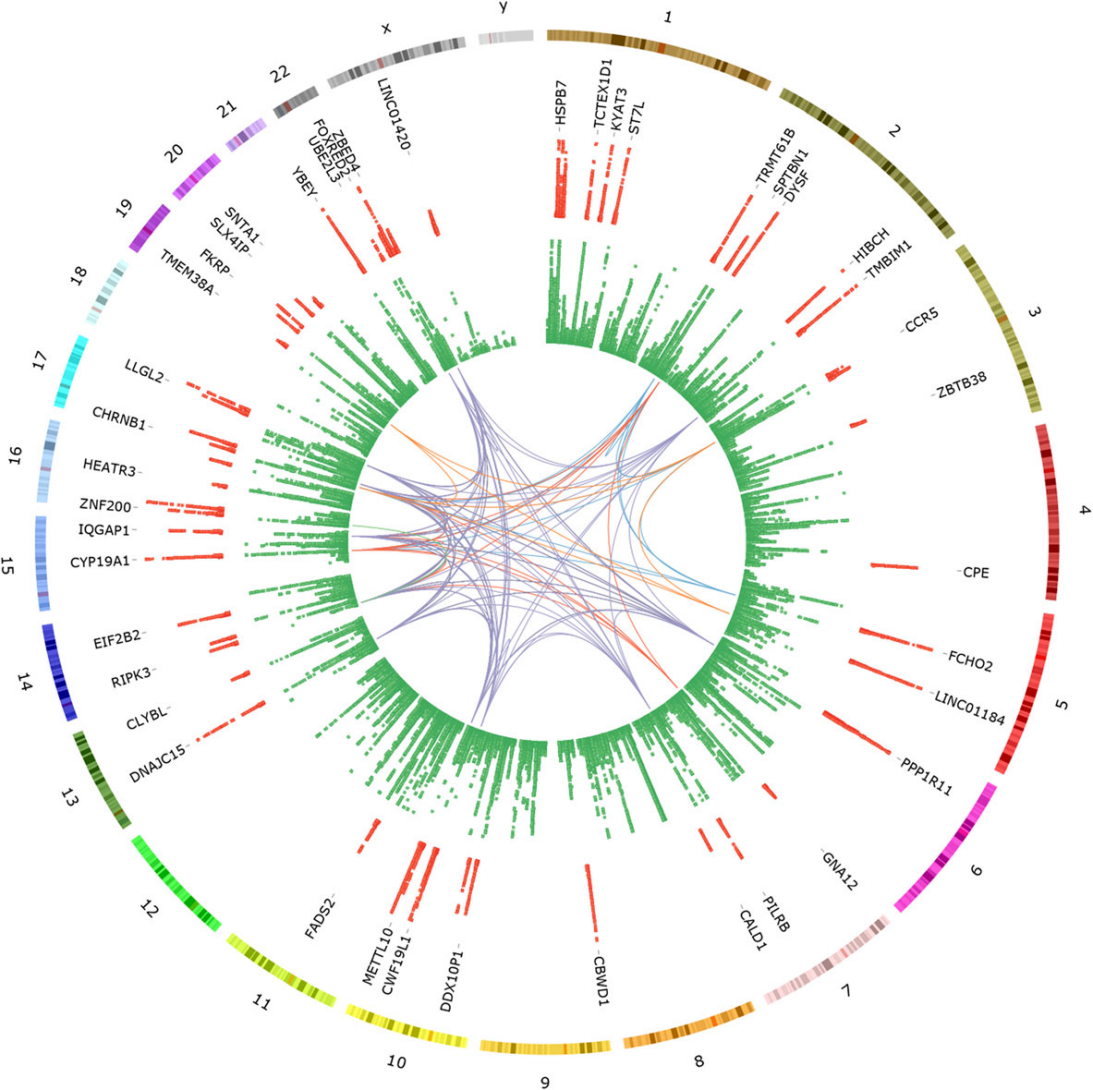
CIRCOS plot [20] displaying (from outside to inside) chromosome numbers, chromosome ideograms, scatterplot complete with gene labels representing the osteoclast eQTL associations presented in Table 1 and Additional file 1: Table S2 (red), scatterplot representing the eBMD GWAS results (green) and gene ontology (GO) biological process groupings relevant to osteoclast biology. Osteoclast eQTL and eBMD GWAS associations are displayed as −log_10_*P* values ranging from 0.01 > *P* > 1.0 × 10^−15^ (osteoclast eQTL) and 0.001 > *P* > 1.0 × 10^−50^ (eBMD GWAS). GO biological process groupings displayed include membrane organisation (blue), regulation of cell migration (red), regulation of catalytic activity (purple), cation transport (orange) and I-kappaB kinase/NF-kappaB signalling (green)

After correction for multiple testing, we observed a total of 69 significant eQTL associations for the eBMD GWAS variants (Table 1, Fig. 1 and Additional file 1: Table S2), with 61 of the variants associated with the expression of 68 genes. Since variants with regulatory effects would be expected to be located in close proximity to their respective eQTL-gene (eGene) transcription start site (TSS), we examined this in our data. Of the 69 eQTL associations, 51 (74%) were for variants located within 150 kb of the eGene TSS, with the average distance being 154.6 kb. The eQTL associations were also stronger for variants located in close proximity to the associated gene, with the 10 strongest eQTL associations identified all representing variants located within 155 kb of the associated eGene TSS. eQTL associations were observed for 8 of the genes identified in a previous smaller study [3], including *PIGV*, *ST7L*, *TRMT61B*, *DYSF*, *FADS2*, *GLDN*, *CYP19A1* and *IQGAP1*. Many of the eQTL variants demonstrated significant associations with expression of more than one gene, including rs11677953 (*RP11-378A13.1* and *TMBIM1*), rs2432236 (*CTD-2376I4.1*, *CTD-2376I4.2* and *FCHO2*), rs42916 (*LINC01184* and *SLC12A2*), rs11814082 (*DDX10P1* and *ZNF438*), rs7147775 (*EIF2B2* and *ACYP1*), rs2899472 (*GLDN* and *CYP19A1*) and rs12325187 (*ZNF200* and *ZNF174*).

**Table 1:** Genetic loci demonstrating co-localised eBMD GWAS and osteoclast *cis*-eQTL associations

Variant	Location	EA	OA	EAF	*β*_GWAS_	Gene	Expression^a^	Distance to TSS	*P*_eQTL_	*β*_eQTL_
rs4683184	chr3:46146215	G	A	0.37	− 0.01	*CCR5*	22.96 ± 7.33	− 224,638	2.06E−04	− 0.47
rs13072536	chr3:52827195	T	A	0.22	0.02	*SFMBT1*	2.87 ± 0.59	− 219,556	1.43E−04	0.50
rs1991431	chr3:141414608	A	G	0.44	− 0.02	*ZBTB38*	7.13 ± 1.57	90,395	7.68E−05	0.44
rs1550270	chr4:165340648	C	T	0.33	0.02	*CPE*	4.64 ± 3.7	− 20,546	7.29E−06	0.51
rs798545	chr7:2722752	T	C	0.23	0.01	*GNA12*	21.9 ± 4.75	− 121,573	7.94E−05	0.53
rs2551769	chr7:135453583	A	G	0.28	0.01	*CALD1*	1.23 ± 1.05	709,328	1.38E−05	− 0.58
rs11245388	chr10:124850559	T	G	0.47	− 0.01	*METTL10*	1.06 ± 0.19	58,688	1.47E−11	0.72
rs3212240	chr14:24341692	C	T	0.43	− 0.01	*RIPK3*	4.51 ± 1.07	1646	1.47E−06	− 0.55
rs7147775	chr14:75203508	G	T	0.44	− 0.02	*EIF2B2*	4.62 ± 0.58	200,597	2.16E−09	− 0.62
rs7147775	chr14:75203508	G	T	0.44	− 0.02	*ACYP1*	1.8 ± 0.56	134,024	4.75E−06	0.55
rs11073930	chr15:90503480	G	C	0.46	− 0.02	*IQGAP1*	66.34 ± 9.26	115,262	1.46E−10	0.66
rs1736213	chr17:17231214	T	G	0.43	0.01	*FLCN*	4.06 ± 0.89	− 5975	1.82E−09	− 0.54
rs1661725	chr17:75564053	C	T	0.44	0.01	*LLGL2*	0.7 ± 0.24	38,973	9.98E−07	− 0.55
rs77420750	chr19:15868934	A	C	0.31	0.01	*TMEM38A*	0.49 ± 0.29	− 792,194	1.43E−04	0.45
rs314675	chr19:46692822	C	T	0.1	− 0.02	*FKRP*	1.45 ± 0.27	− 53,224	5.88E−06	0.92
rs73066226	chr19:58496846	C	T	0.17	0.01	*UBE2M*	22.85 ± 3.39	− 62,115	1.71E−06	0.66
rs5754387	chr22:21620414	C	G	0.17	− 0.02	*UBE2L3*	20.1 ± 2.88	70,967	1.82E−05	0.46
rs2294358	chr22:36375211	C	G	0.07	0.04	*FOXRED2*	2.44 ± 1.37	− 131,893	1.55E−06	1.11
rs932536	chr22:49866824	A	G	0.13	− 0.02	*ZBED4*	5.67 ± 0.67	12,982	8.39E−06	0.71
rs5770908	chr22:50439289	A	G	0.29	− 0.02	*PPP6R2*	10.17 ± 1.8	95,985	1.36E−04	0.41
rs5914035	chrX:56981783	C	T	0.23	0.02	*LINC01420*	13.14 ± 2.09	252,542	4.91E−06	0.62

*EA* effect allele, *OA* other allele, *EAF* effect allele frequency (derived from the osteoclast eQTL cohort), *eBMD* estimated BMD, *TSS* transcription start site; variant locations derived from dbSNP build 150 (GRCh38/hg38), β_GWAS_ values are relevant to the effect allele and were obtained from Morris et al. [19], β_eQTL_ values are given as the normalised effect size on gene expression for the effect allele. eQTL associations are significant using a multiple-testing corrected FDR of 5%

^a^Expression levels are stated as mean reads per kilobase million (RPKM) ± standard deviation

### Co-localisation analysis

Since it is possible for independent GWAS and eQTL associations to exist within a genetic locus, we decided to assess co-localisation of eBMD GWAS and osteoclast eQTL association signals for each of the 69 significant eQTL loci using the coloc package in R [21]. If the association signals within the two datasets are shown to co-localise, it can be interpreted as strong evidence that the GWAS association is mediated through regulatory effects on the eQTL gene. Evidence of co-localisation of GWAS and eQTL association signals was identified (posterior probability > 50%) in 19 of the 69 loci, including *CCR5* (82.1%), *ZBTB38* (83.3%), *CPE* (54.9%), *GNA12* (50.1%), *CALD1* (91.3%), *METTL10* (96.6%), *RIPK3* (89.8%) (Additional file 1: Fig. S1), *EIF2B2* (65.9%), *ACYP1* (83.4%), *IQGAP1* (75.2%), *FLCN* (95.6%), *LLGL2* (78.1%), *TMEM38A* (71.1%), *FKRP* (84.0%), *UBE2M* (82.6%), *UBE2L3* (79.5%), *FOXRED2* (55.0%), *PPP6R2* (73.1%) and *LINC01420* (87.2%). The eBMD GWAS association signal at 14q24.3 led by rs7147775 demonstrated strong evidence for co-localisation with eQTL effects for both the *EIF2B2* and *ACYP1* genes (Additional file 1: Fig. S2). Co-localisation analysis was also performed using a newer, but as yet less widely evaluated version of the coloc R functions, named coloc2, which is thought to more fully address allelic heterogeneity when present in the data [22]. Apart from *CPE* (3.0%) and *LLGL2* (1.0%), all of the genetic loci demonstrating co-localisation in the coloc analysis also presented with co-localisation using coloc2 (posterior probabilities 98.3–100%). Two additional co-localised genetic loci were identified by coloc2, *SFMBT1* (98.2%) and *ZBED4* (99.1%). eBMD GWAS and osteoclast eQTL results for all co-localised loci are presented in Table 1, with those demonstrating independent (non-co-localised) association signals presented in Additional file 1: Table S2. An example of a locus with independent eBMD GWAS and osteoclast eQTL association signals, *LINC01184* (posterior probability of co-localisation < 0.01% using coloc [21] and coloc2 [22]), is displayed in Additional file 1: Fig. S3.

### Tissue-shared and osteoclast-specific eQTL associations

To establish the proportion of the identified eQTL associations that have supporting evidence from other tissue types and to identify those that are potentially osteoclast-specific, we analysed each of the 69 eQTL associations for evidence of replication in the 53 tissues of the GTEx V7 dataset [1]. Only GTEx tissues with > 70 samples were included in the analysis, with a multiple-testing corrected false discovery rate (FDR) threshold of 0.05 used to identify significant associations. Of the 69 eQTL associations, 45 (65.2%) demonstrated evidence of association in at least one GTEx tissue, leaving 24 (34.8%) as potentially specific to osteoclasts. These included the eQTL associations for the genes *HSPB7*, *NBPF3*, *ZNF593*, *PIGV*, *KYAT3*, *HIBCH*, *CCR5*, *CPE*, *SLC12A2*, *PPP1R11*, *CALD1*, *CBWD1*, *ZNF438*, *RP11-517P14.2*, *GLDN*, *ZNF174*, *NLRC3*, *HEATR3*, *RP11-104 N10.2*, *TMEM38A*, *UBE2M*, *SLX4IP*, *SNTA1* and *LINC01420*.

### Summary-data-based Mendelian Randomisation analysis

In order to identify genes whose expression levels in osteoclasts are associated with the eBMD trait, we performed an integrative analysis of the eBMD GWAS and osteoclast eQTL datasets using the Summary-data-based Mendelian Randomisation (SMR) software [23]. A total of 1070 genes with an eQTL association significant at *P* < 5 × 10^−8^ were identified and included in the analysis. Using a multiple-testing corrected significance threshold of *P*_SMR_ < 4.7 × 10^−5^, the SMR test identified significant associations for 53 genes (Additional file 1: Table S3). Heterogeneity (*P*_HEIDI_ < 0.05) was detected for 52 of these, leaving a single locus *TULP4* as a strong candidate for harbouring shared genetic effects (i.e. pleiotropy) on gene expression in osteoclasts and eBMD (Additional file 1: Fig. S4). Expression of *TULP4* was found to be positively associated with eBMD (*β*_SMR_ = 0.02, Additional file 1: Table S3).

### Enrichment of osteoporosis risk variants within osteoclast eQTL

**Fig. 2:**
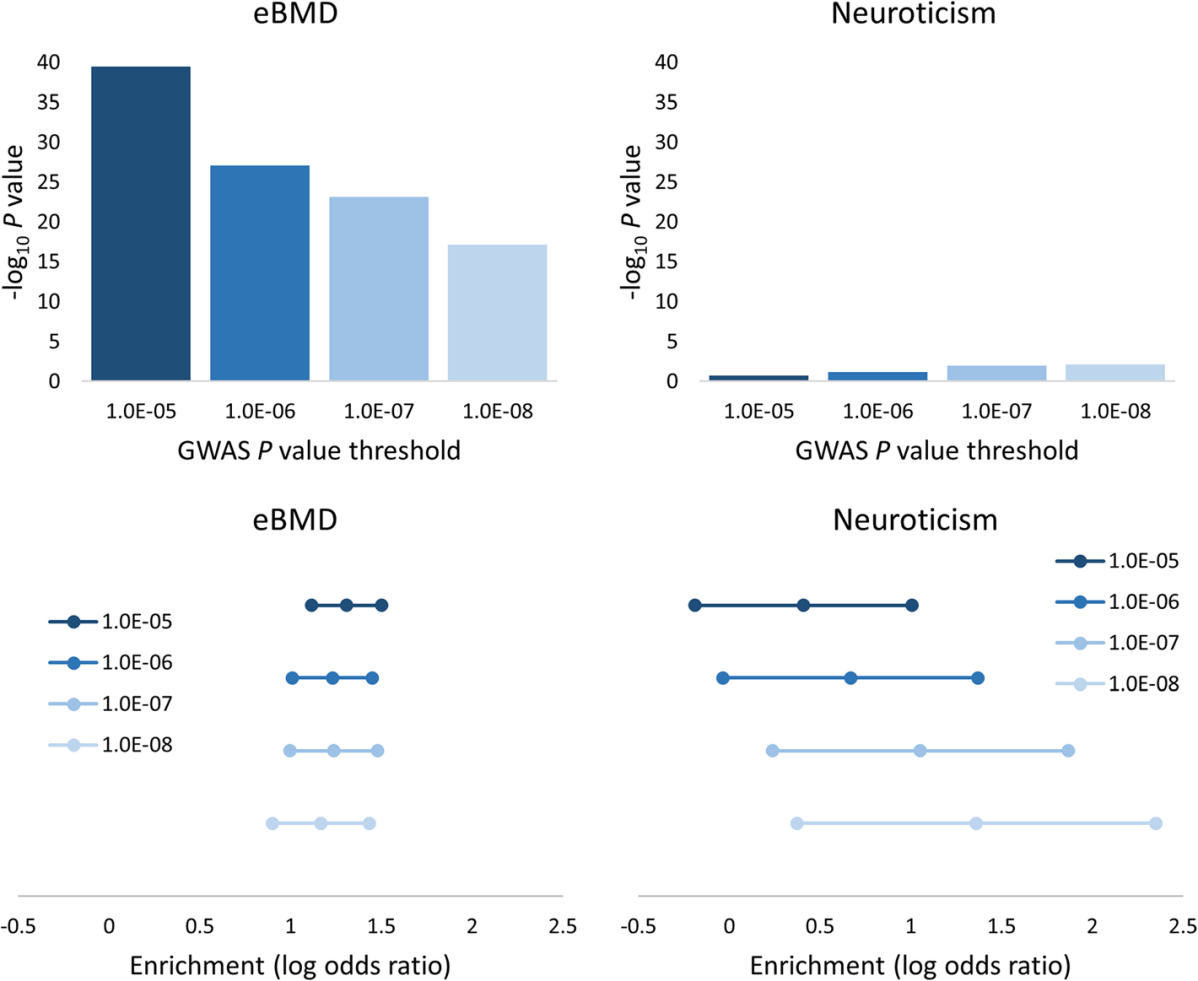
Analysis of the eBMD GWAS and osteoclast eQTL datasets using the GARFIELD software [24] demonstrated significant enrichment of osteoporosis risk variants among osteoclast eQTL across four GWAS *P* value thresholds (*P* < 1 × 10^−5^, 1 × 10^−6^, 1 × 10^−7^ and 1 × 10^−8^), with the enrichment results for the trait neuroticism [25] included for comparison. The upper panels present the −log10 enrichment *P* values for each threshold, while the lower panels present the natural logarithm of the odds ratios for each threshold with 95% confidence intervals

We used the GWAS Analysis of Regulatory or Functional Information Enrichment with LD correction (GARFIELD) software [24] to determine whether osteoporosis risk variants in the eBMD GWAS summary result dataset are enriched among high-confidence osteoclast eQTL variants identified using a multiple-testing corrected FDR threshold of 0.05. Using eBMD GWAS *P* value thresholds of *P* < 1 × 10^−5^, 1 × 10^−6^, 1 × 10^−7^ and 1 × 10^−8^, we observed significant enrichment of osteoporosis risk variants within osteoclast eQTL (*P* = 3.25 × 10^−40^, 8.83 × 10^−28^, 6.93 × 10^−24^ and 7.79 × 10^−18^, respectively, Fig. 2). As a comparison, we also performed enrichment analysis using GWAS results from a similarly powered study for a trait not considered to be related to bone biology, neuroticism [25]. We observed nominally significant enrichment of neuroticism GWAS variants within the osteoclast eQTL dataset at the *P* < 1 × 10^−7^ and 1 × 10^−8^ thresholds (*P* = 0.01 and 0.007, respectively, Fig. 2); however, these were not significant after correction for multiple testing.

### Analysis of *Ripk3* knockout mice

**Fig. 3:**
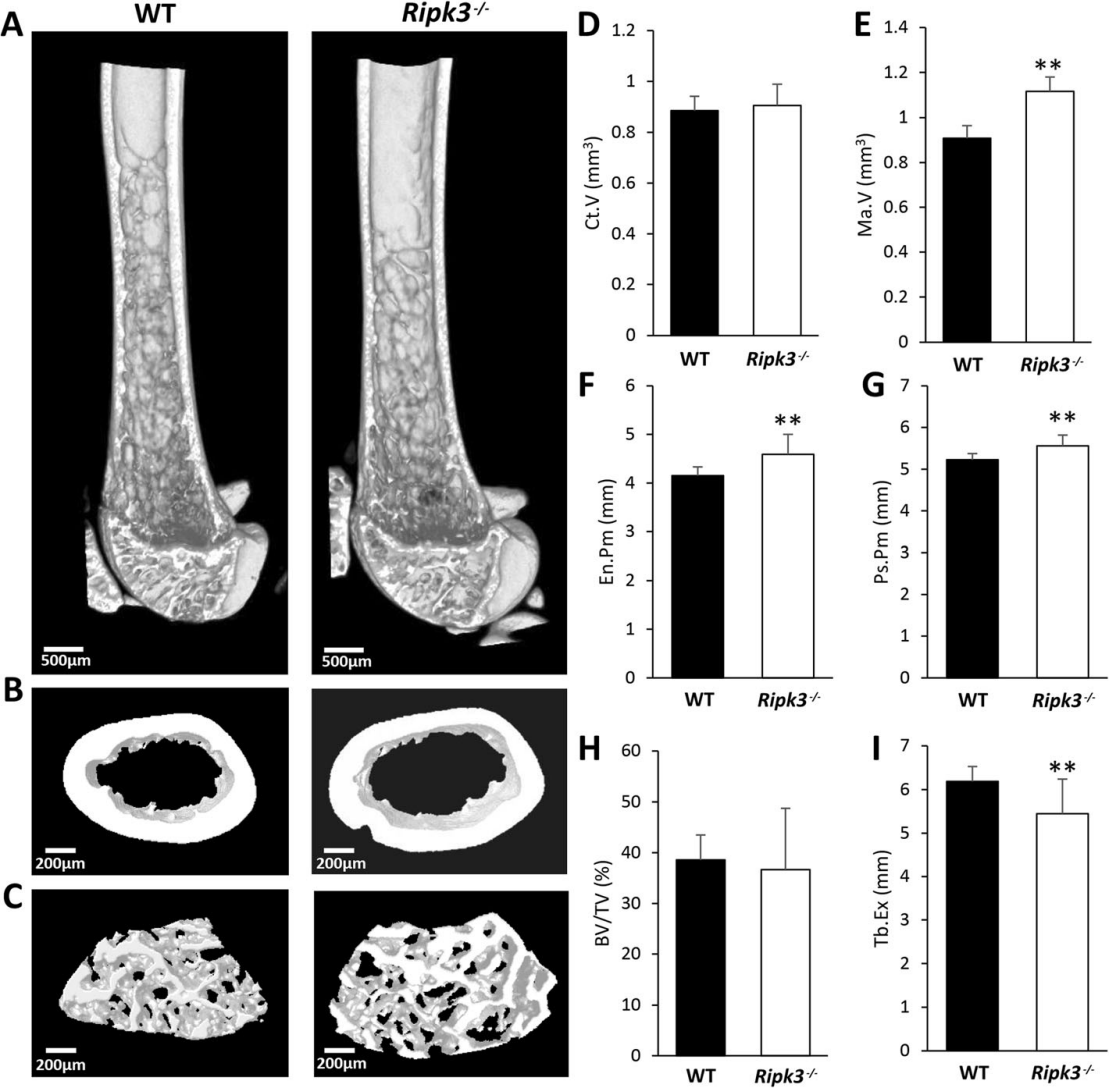
Micro-CT assessment of the distal femur from 15-week-old male *Ripk3*^*−/−*^ and WT mice. Representative 3D images of the distal femur demonstrate the following: **a** reduced extension of the trabecular network into the diaphysis, **b** expansion of the medullary cavity and **c** increased size of the trabecular bone compartment with no change in trabecular bone density in the *Ripk3*^*-/-*^ mice. Quantitative analysis of micro-CT parameters (mean + standard deviation) displays the following: **d** cortical volume, **e** bone marrow volume, **f** endosteal perimeter, **g** periosteal perimeter, **h** trabecular bone volume fraction and **i** trabecular extension in the *Ripk3*^*−/−*^ mice relative to WT mice. *N* = 5 for each group; WT, wildtype. **P < 0.01

Among the genes identified in this study as potentially relevant to osteoporosis is *RIPK3*. According to data available in GTEx V7, the eBMD GWAS variant rs3212240 is an eQTL for *RIPK3* only in sun-exposed skin (*P* = 2.0 × 10^−5^); however, data from this study suggests that it is also relevant to bone cells. The *C* allele at rs3212240, associated with a lower eBMD measurement, was found to be associated with reduced expression of the *RIPK3* gene in our osteoclast eQTL dataset (Table 1). Because of the strong evidence for co-localisation of eBMD GWAS and osteoclast eQTL association signals within this locus, combined with the known role of *RIPK3* in cellular necroptosis in response to the tumour necrosis factor (TNF)-alpha family of death-inducing cytokines and potential involvement in bone resorption in osteoporosis [26], we performed in-depth skeletal phenotyping of *Ripk3*-deficient mice. Analysis of the distal femur of 15-week-old male *Ripk3*^*−/−*^ mice using micro-CT revealed increased subperiosteal perimeter, increased endosteal perimeter and increased bone marrow volume compared to wildtype (WT) animals (Fig. 3). There was no difference in cortical bone volume or trabecular indices between *Ripk3*^*−/−*^ and WT mice (Additional file 1: Fig. S5). However, the extent of trabecularisation along the diaphysis was significantly reduced in the *Ripk3*^*−/−*^ mice (Fig. 3a).

**Fig. 4:**
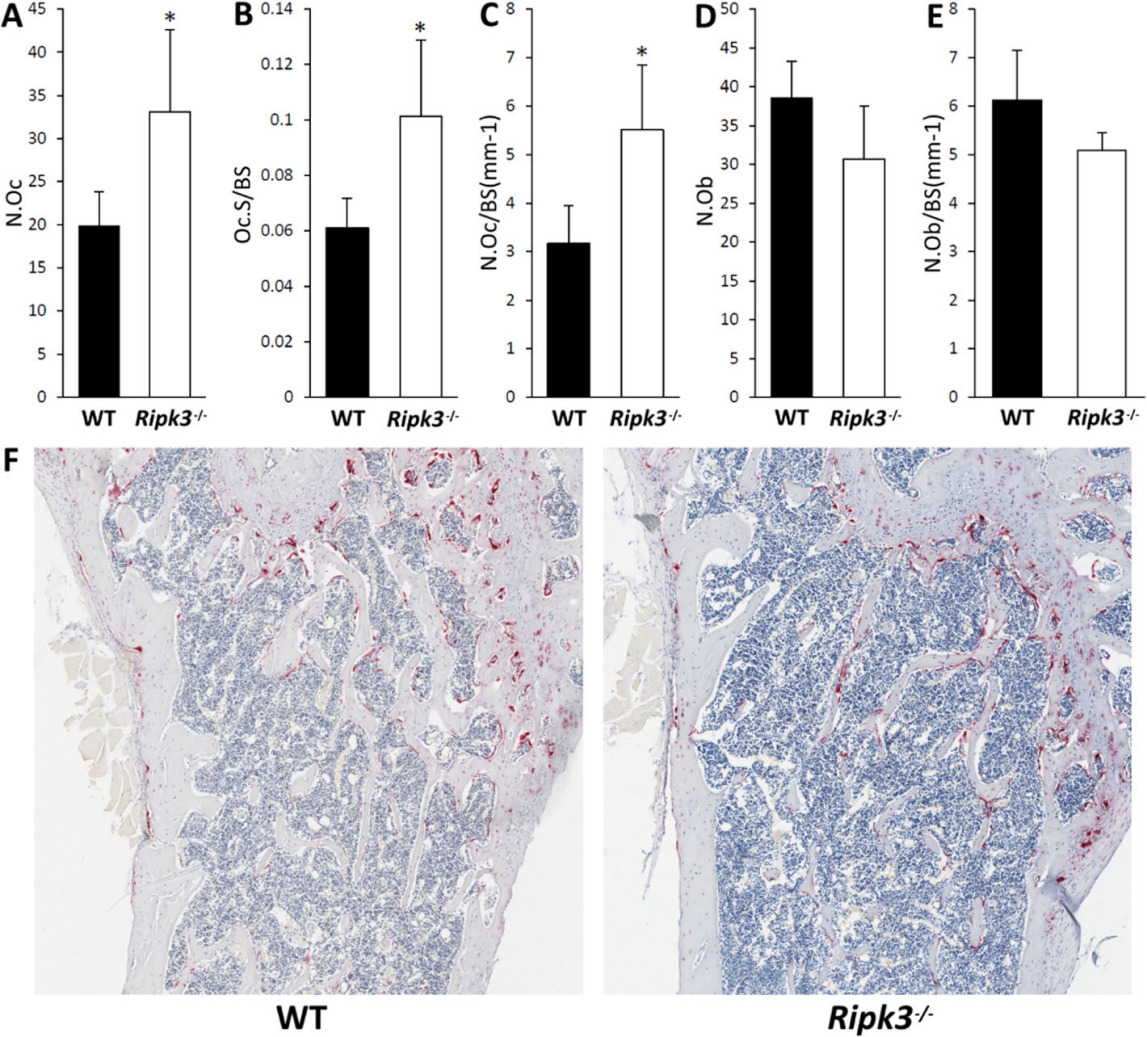
Quantitative histomorphometric analysis of femora from 15-week-old male WT and *Ripk3*^*−/−*^ mice (mean + standard deviation). **a** Osteoclast number (N.Oc). **b** Osteoclast surface relative to bone surface (Oc.S/BS). **c** Number of osteoclasts relative to bone surface (N.Oc/BS(mm^−1^)). **d** Osteoblast number (N.Ob). **e** Number of osteoblasts relative to bone surface (N.Ob/BS(mm^−1^)). **f** Representative low-power images (× 40 magnification) of TRAP-stained sections of the femur just below the growth plate. *N* = 4 and 5 for the WT and *Ripk3*^*−/−*^ groups, respectively; WT, wildtype

Decalcified bone sections were stained for TRAP activity and with haematoxylin and eosin (H&E) to further characterise the cellular features of the *Ripk3*^−/−^ mice by histomorphometry (Fig. 4a–f). Analysis of the TRAP-stained bone sections revealed a significant increase in the total number of osteoclasts, number of osteoclasts relative to bone surface and osteoclast surface relative to bone surface in the *Ripk3*^−/−^ bones relative to those from the WT mice (Fig. 4a–c). No significant differences in osteoblast parameters were observed between the *Ripk3*^−/−^ and WT bones (Fig. 4d, e). These results suggest that *Ripk3*^*−/−*^ mice present with increased osteoclast number in vivo.

## Discussion

In this study of human osteoclast-like cells, we have identified a large number of genetic regulatory effects for osteoporosis risk variants. The majority of the eQTLs identified in this study are located in close proximity to their effector gene TSS, consistent with findings from the GTEx project [27]. A significant proportion (34.8%) of the eQTL associations identified in this study are potentially osteoclast-specific when compared with data from the 53 tissues of the GTEx V7 dataset [1], demonstrating the value of performing such investigations in disease-relevant cell/tissue types. We also found that high-confidence osteoclast eQTLs are strongly enriched for osteoporosis risk variants, with an average 3.5-fold enrichment seen across the four GWAS significance thresholds tested.

An osteoporosis-relevant eQTL association was identified in this study for the eBMD GWAS variant rs3212240, which was found to be significantly associated with expression of the *RIPK3* gene, a member of the receptor-interacting protein (RIP) family of serine/threonine protein kinases. Co-localisation analysis of the locus suggested that the eBMD GWAS and osteoclast eQTL association signals may be driven by the same causal variant. Analysis of *Ripk3*^*−/−*^ mice revealed a variety of bone effects, primarily characterised by expansion of the femoral shaft with reduced extension of the trabecular network and an increase in marrow volume. The effects observed on trabecular extension could potentially account for the association with eBMD in humans, a phenotype derived using quantitative ultrasound measurements at the heel and hence primarily influenced by bone structural parameters including the orientation of the trabecular bone [28], as opposed to BMD. The lead eBMD GWAS variant from the human *RIPK3* locus, rs3212240, does not demonstrate even nominal associations with total body [14], femoral neck or lumbar spine [18] BMD derived using dual-energy X-ray absorptiometry. The product of the *RIPK3* gene is a component of the TNF receptor 1 signalling complex and has an established role in necroptosis [29, 30], a type of programmed cell death thought to cause inflammation through the release of intracellular factors [31]. Our histomorphometric analysis of the *Ripk3*^*−/−*^ bones revealed increased numbers of osteoclasts relative to WT bones, suggesting that this gene may have a role in osteoclast necroptosis and that loss of this mechanism may lead to an excess of osteoclasts. If this is the case, it could potentially explain why the *C* allele at rs3212240 is associated with reduced expression of the *RIPK3* gene in our osteoclast eQTL dataset and a reduced eBMD [19]. The variant rs3212240 is located ~ 1.5 kb upstream of the *RIPK3* TSS and is in moderate linkage disequilibrium (LD) (*r*^2^ = 0.50) with a nearby body height GWAS variant (rs1950500) [32]. It is also in moderate LD (*r*^2^ = 0.79) with a missense variant in an adjacent gene, *NFATC4* (rs2229309), and we cannot rule out the possibility that the association at this locus with eBMD is partially mediated through the effects of this variant. However, rs2229309 is classified as benign by various pathogenicity prediction algorithms [33–35]. Considering the strong regulatory effect on *RIPK3* demonstrated for rs3212240, combined with the altered bone phenotypes observed in *Ripk3*^*−/−*^ mice, we consider that the association at this locus with eBMD is likely to be mediated through regulatory effects on the *RIPK3* gene.

A novel eQTL association relevant to osteoporosis was identified in this study for the *CPE* gene. This locus also presented with evidence for co-localisation of eBMD GWAS and osteoclast eQTL association signals using the coloc package [21] and was identified as a significant locus in the SMR analysis. Of note, the association seen in our data between the eBMD GWAS variant rs1550270 and expression of *CPE* was not observed in any of the 53 tissues of the GTEx V7 dataset [1]. The product of the *CPE* gene, carboxypeptidase E, is a prohormone-processing enzyme that cleaves carboxy-terminal basic residues from neuropeptide intermediates and peptide hormones to produce mature bioactive products, including insulin [36]. The *CPE* gene has been strongly implicated in diabetes, with mutation in the human *CPE* gene identified as the likely cause of a genetic disease characterised by obesity, type 2 diabetes, intellectual disability and hypogonadotrophic hypogonadism [37]. A deactivating mutation in the murine *Cpe* gene also results in a pronounced obesity-diabetes syndrome [38]. *Cpe*-knockout mice present with obesity and reduced BMD with increased plasma levels of osteocalcin and carboxy-terminal collagen crosslinks, indicating the presence of increased bone turnover [39]. Expression of receptor activator of nuclear factor kappa-B ligand (RANKL) was found to be elevated approximately 2-fold relative to osteoprotegerin in the femora of *Cpe*-deficient mice, potentially leading to increased osteoclast activity [39]. It has also been established that expression of *Cpe* is highly upregulated in mouse osteoclasts compared to their precursor cells and that this upregulation is associated with increased formation of TRAP-positive multinucleated osteoclasts and expression of key genes involved with osteoclastogenesis [40]. Our osteoclast eQTL data indicates that the human *CPE* gene has a role in bone density regulation and highlights this gene as a potential pleiotropic locus relevant to diabetes and osteoporosis.

We observed some interesting eQTL effects in this study for several genes implicated in monogenic disorders. An example of this is the *FBN2* gene, encoding fibrillin 2, which is an extracellular protein that is a major component of connective tissue microfibrils. Mutation in this gene has been identified as the cause of congenital contractural arachnodactyly (CCA) [41], a disorder phenotypically similar to Marfan syndrome (caused by mutation in the related gene *FBN1*). CCA is characterised by a variety of skeletal abnormalities including congenital contractures, arachnodactyly, deformities of the chest wall, scoliosis and elongated limbs. Another example is the gene *FLCN*, encoding folliculin, which was identified as a significant locus in the SMR analysis and demonstrated strong evidence for co-localisation of eBMD GWAS and osteoclast eQTL association signals. Folliculin has recently been shown to regulate osteoclastogenesis through metabolic effects on oxidative phosphorylation and purine metabolism [42]. Induced deletion of the murine *Flcn* gene in osteoclast precursors was found to result in severe osteoporosis caused by excess osteoclastogenesis [42]. Mutation in the human *FLCN* gene has been identified as the cause of Birt-Hogg-Dubé syndrome [43], an autosomal dominant condition characterised by early onset renal tumours, hair follicle hamartomas, pulmonary cysts and spontaneous pneumothoraces. However, we are not aware of any significant bone or skeletal phenotypes that have been reported in instances of this condition to date.

The SMR analysis identified a significant association for the *TULP4* gene, with the HEIDI test suggesting that the locus may exhibit pleiotropic effects on *TULP4* expression and eBMD. The product of the *TULP4* gene has not been extensively characterised with regard to a role in bone biology; however, it is thought to be a cytoplasmic protein characterised by a large amino terminus containing a WD40 repeat region and a suppressor of cytokines signalling domain [44]. *TULP4* has been implicated in the formation of orofacial clefts [45] and is a body height GWAS locus [32, 46]. Mutation in the related gene *WDR35* has been implicated in cranioectodermal dysplasia, also known as Sensenbrenner syndrome, an autosomal recessive condition characterised by craniofacial and skeletal abnormalities [47]. The top eQTL variant identified for *TULP4* is rs341106, which is only in weak LD with the lead eBMD GWAS variant from the locus rs12206717 (*r*^2^ = 0.02 in Europeans [48]). rs12206717 is a missense variant in exon 9 of *TULP4*, which causes a change of amino acid 522 from serine to asparagine. It is possible that there are 2 independent association signals for eBMD arising from the locus, one potentially causing a structural change in the *TULP4* protein and the other influencing expression of the gene.

It is worth noting that the differentiated osteoclast-like cells used in this study likely express a smaller panel of genes when compared to complex tissues containing a mixture of cell types such as those used in the GTEx project. When this is considered in the context of a relatively modest sample size, it potentially explains why a greater number of co-localised genetic loci were not identified in this study. It is also possible that the eBMD GWAS data are primarily reflective of bone accrual and osteoblast function and that resorption and osteoclast activity are not the primary drivers of those results. It should also be noted that the threshold used to identify co-localised loci (posterior probability > 50%) is merely a guideline and that probabilities below this value do not necessarily indicate independent association signals, particularly when considering the sample size of the osteoclast eQTL cohort. The SMR software may also be susceptible to small-sample biases, which could potentially explain the large proportion of significant HEIDI test scores seen in the study.

## Conclusions

We have used a unique osteoclast-specific eQTL dataset to identify a number of genetic regulatory effects relevant to osteoporosis. Approximately one third of these regulatory effects were not seen in the 53 tissues of the GTEx V7 dataset [1] and are potentially osteoclast-specific. A number of the genes highlighted in this study present strongly as having a potential role in bone biology, including *CPE*, *FBN2*, *FLCN* and *RIPK3*, and our results support the growing body of evidence suggesting that the *CPE* gene may exert pleiotropic effects on type 2 diabetes and osteoporosis. We performed thorough skeletal phenotyping of *Ripk3*-deficient mice, which presented with altered bone micro-architectural characteristics potentially driven through effects on osteoclast necroptosis. The results from this study have identified a number of potential effector genes for GWAS loci relevant to osteoporosis, which will help target future translational studies.

## Materials and methods

### Subject recruitment and generation of osteoclast-like cells

Details of the recruitment process and cell culture procedures used in this study have been described previously [3]. Briefly, the osteoclast eQTL study cohort comprises 158 women aged 30–70 years with self-reported European ancestry who attended the Bone Density Unit at Sir Charles Gairdner Hospital in Western Australia for a dual-energy X-ray absorptiometry BMD scan in 2016 (Hologic, Bedford, MA, USA). Exclusion criteria used during recruitment included presence of medical conditions or use of medications that are likely to influence osteoclastic bone resorption or the process of osteoclastogenesis. Osteoclast-like cells were generated using the conventional procedure; peripheral blood mononuclear cells were first isolated from blood samples obtained from each individual by density gradient centrifugation using protocols well established in our laboratory [3, 49]. These cells were cultured in triplicate for 2 days in α-MEM supplemented with 25 ng/ml macrophage colony stimulating factor (M-CSF), then for a further 12 days in α-MEM supplemented with 25 ng/ml M-CSF and 100 ng/ml RANKL while osteoclastogenesis occurred. The osteoclastic character of the cultures was verified by staining for tartrate-resistant acid phosphatase (TRAP) as described previously [3] and by gene expression profiling (Additional file 1: Fig. S6).

### Nucleic acid extraction

Genomic DNA for each participant was extracted from whole blood using the QIAamp DNA Blood Mini Kit (QIAGEN) according to the manufacturer’s instructions. At day 14 of culture, RNA and DNA were harvested from each set of triplicate osteoclast-like cell cultures using the AllPrep DNA/RNA Mini Kit (QIAGEN) according to the manufacturer’s instructions, with on-column DNase digestion for the RNA fraction. High-quality RNA was obtained, with all samples recording RNA integrity numbers (RINs) ≥ 9.7.

### Genotyping and imputation

Genome-wide array genotyping was performed on the genomic DNA samples using the Illumina Infinium OmniExpress-24 BeadChip array. QC criteria applied to the genotype data included removal of individuals with a call rate < 90%, variants that were monomorphic, unmapped, had a MAF < 5%, Hardy-Weinberg equilibrium *P* < 5 × 10^−8^ or call rate < 90%, leaving 572,898 variants for imputation. Genotype imputation was then performed by the Sanger Imputation Service using the Haplotype Reference Consortium (HRC) release 1.1 reference panel [50]. Any variants with an IMPUTE2 info score < 0.4 were removed from the imputed dataset. Relatedness testing and principal components analysis was performed on the genotype dataset using Plink v1.9 [51], with 10 principal components generated for use as covariates in the eQTL analysis to correct for population stratification.

### Generation and processing of gene expression data

Transcriptome-wide quantitation of gene expression was performed on the osteoclast RNA samples using 50 bp single-end RNA-Seq on an Illumina HiSeq 2500. Raw read counts were generated for each gene (GENCODE v25), while those displaying a read count < 1 per million or expressed in < 10 individuals were removed. Trimmed mean of *M* value (TMM) normalisation and correction for total read count by conversion to counts per million (CPM) was performed on the gene expression data using the edgeR package [52]. Reads per kilobase per million (RPKM) values were also calculated for each gene using edgeR [52] to enable comparison of expression levels between different genes.

### eQTL association analysis

The top genetic variant at each of the 1103 recently identified independent association signals for eBMD [19] was investigated for *cis*- (local) eQTL effects in the osteoclast samples. The eQTL analysis was performed on the TMM-normalised CPM gene expression values using the FastQTL software [53], which performs linear regressions between genotypes and gene expression values. Quantile normalisation was implemented using FastQTL (based on the *rntransform* function of the GenABEL package [54]) to ensure the gene expression values were normally distributed with a mean of 0 and standard deviation of 1. Only variants with a MAF ≥ 5% were included in the analysis, which was adjusted for the covariates patient age, RNA-Seq batch and 10 genomic principal components. Each variant was tested for association with the expression of any gene with a TSS located within a 1 Mb window on either side of the variant, consistent with the methods used by the GTEx project. Correction for multiple testing was performed using the Benjamini-Hochberg procedure [55], utilising a FDR of 5%.

### Co-localisation analysis

To assess the probability that eBMD GWAS and osteoclast eQTL association signals residing in the same locus share the same causal variant, we performed a co-localisation analysis of the two datasets using the coloc package in R (default settings) [21]. This software uses a Bayesian framework to calculate posterior probabilities for 5 different scenarios (hypotheses) regarding the presence and sharing of causal variants between two genetic association datasets using summary statistics. A posterior probability of > 50% for hypothesis H_4_ for a specific locus (association with trait 1 and trait 2, one shared variant) indicates that co-localisation of the association signals in the two datasets due to a shared causal variant is the most likely of the 5 scenarios. We also performed co-localisation analysis using an updated version of the coloc software, named coloc2 [22]. Coloc2 performs alignment of the GWAS and eQTL summary results for each *cis*-eQTL region as a pre-processing step and includes optional changes implemented in the gwas-pw algorithm [56].

### Summary-data-based Mendelian Randomisation analysis

To further characterise functionally relevant genes for the eBMD GWAS associations and to identify potential pleiotropic effects on gene expression and eBMD, we performed integration of the complete set of eBMD GWAS summary results with the osteoclast eQTL dataset (± 1 Mb) using the SMR software [23]. This package applies the principles of Mendelian randomisation [57] to test for association between gene expression and a trait due to a shared variant at a genetic locus. The software performs an SMR test, which detects dual association signals in the GWAS and eQTL datasets by testing for association between gene expression and the trait of interest at the top associated eQTL for each gene. The software also performs a heterogeneity in dependent instruments (HEIDI) test, which compares the association signals for nearby co-inherited markers in the GWAS and eQTL datasets. A significant HEIDI test indicates heterogeneity in the association profiles of the two datasets, thereby suggesting that the association signals seen in each dataset are less likely to be driven by the same causal variant. The osteoclast eQTL cohort genotype dataset was used as the reference panel in these analyses for estimation of LD, and only genes with at least 1 *cis*-eQTL association at *P* < 5 × 10^−8^ were included (*N* = 1070). A Bonferroni multiple-testing corrected significance threshold of *P* < 4.7 × 10^−5^ was used for the SMR test (*P*_SMR_), while a conservative significance threshold of *P* < 0.05 was set for the HEIDI test (*P*_HEIDI_) as an indicator of heterogeneity.

### Osteoclast eQTL enrichment analysis

The GARFIELD software [24] was used to test for enrichment of osteoporosis risk variants among high-confidence osteoclast eQTL. GARFIELD performs LD pruning (*r*^2^ > 0.1) of GWAS association results to generate an independent set of variants and then integrates this with annotations containing regulatory or functional information. Each variant is annotated to a functional category or regulatory feature if the variant or an LD proxy (*r*^2^ > 0.8) is part of that annotation group. Fold enrichment is calculated using odds ratios at specified GWAS *P* value thresholds with significance assessed using a generalised linear model while accounting for MAF, distance to nearest TSS and number of LD proxies. A custom annotation category was generated containing all significant osteoclast eQTL associations identified at FDR 5%. Enrichment of eBMD GWAS variants in this annotation was assessed at four GWAS significance thresholds: *P* < 1 × 10^−5^, 1 × 10^−6^, 1 × 10^−7^ and 1 × 10^−8^. The UK10K variant set was used as the reference population for these analyses, with correction for multiple testing performed using the Bonferroni method.

### X-ray microcomputed tomography analysis of mouse bones

*Ripk3*-deficient mice were generated and phenotyped as described previously [58]. Femora were cleaned of soft tissue and fixed in 10% neutral buffered formalin for 48 h, followed by 24 h immersion in phosphate-buffered saline (PBS). Five pairs of 15-week-old male *Ripk3*^−/−^ mice and WT controls were used for the micro-CT analysis. Fixed femora were immersed in PBS and immobilised in a 2-ml tube prior to scanning of the distal femur using a Skyscan 1176 micro-CT instrument (Bruker, Kontich, Belgium). Scan parameters were as follows: 50 kV, 500 μA, 0.5 mm Al filter, exposure time 1 s, 0.4° rotation step, frame averaging of 2 and pixel resolution 8.89 μm. Scans were reconstructed using NRecon software (Bruker, Kontich, Belgium) using a constant threshold value and then analysed using the CTAn software (Bruker, Kontich Belgium). A trabecular region of interest was defined 0.5 mm below the base of the growth plate and 1 mm in height. A cortical region of interest was defined 3 mm below the growth plate and 1 mm in height. Trabecular extent was defined as the distance from the base of the growth plate to the furthest definable continuous trabecular element. Reported trabecular values were bone volume/tissue volume (BV/TV), trabecular thickness (Tb.Th), trabecular separation (Tb.Sp), trabecular number (Tb.N), trabecular extension (Tb.Ex) and BMD; reported cortical values were cortical thickness (Ct.Th), cortical bone volume (Ct.BV), marrow volume (Ma.V), endocortical perimeter (Ec.Pm), periosteal perimeter (Ps.Pm) and tissue mineral density (TMD). Differences between WT and *Ripk3*^*−/−*^ samples were assessed using an unpaired *t* test.

### Histomorphometric analysis of mouse bones

Mouse femur samples were formalin-fixed, decalcified and embedded in paraffin before being subjected to staining with H&E and a chromogenic TRAP substrate to characterise in vivo osteoblast and osteoclast parameters. Stained bone sections were scanned using a Scanscope XT machine (Aperio) at × 20 objective, with histomorphometric analysis performed using the BioQuant Osteo software (BioQuant). To characterise the trabecular bone, a region of interest located approximately 500 μm below the growth plate at the distal femur and 1 mm in height was defined. For the cortical bone analysis, we used a region located approximately 4 mm below the growth plate with a height of 1 mm. Two sections were analysed for each femur, with the mean of the two sets of measurements used for statistical analysis. Differences between the WT and *Ripk3*^*−/−*^ groups were assessed using an unpaired *t* test.

## Supplementary information

**Additional file 1: Tables S1-S3** and **Fig. S1-S6.**

**Additional file 2.** Review history.

## Data Availability

The GTEx V7 eQTL dataset can be accessed at https://gtexportal.org/home/datasets. The osteoclast eQTL data described in this study relevant to the genes listed in Table 1 are publicly available from the figshare repository [59].
