# Supplementary material for: Characterisation of genetic regulatory effects for osteoporosis risk variants in human osteoclasts
Source: Genome Biol. 2020 Mar 26;21:80. doi: 10.1186/s13059-020-01997-2 (PMC7098081; doi:10.1186/s13059-020-01997-2)
Supplement: Supplementary file 1 — Additional file 1: Tables S1-S3 and Fig. S1-S6. [file 13059_2020_1997_MOESM1_ESM.docx]

**Table S1:** Demographics of the osteoclast eQTL study cohort

| **Demographic variable** | **Mean (SD)** |
| --- | --- |
| Subjects (n) | 158 |
| Age (years) | 57.2 (9.7) |
| Age (range) | 30.5 – 69.8 |
| Height (cm) | 162.7 (6.2) |
| Weight (kg) | 67.7 (13.0) |
| BMI (kg/m^2^) | 25.6 (5.1) |
| Spine BMD (g/cm^2^) | 0.94 (0.15) |
| Spine BMD T-score | -0.96 (1.34) |
| Total hip BMD (g/cm^2^) | 0.84 (0.12) |
| Total hip BMD T-score | -0.80 (0.99) |
| Femoral neck BMD (g/cm^2^) | 0.72 (0.11) |
| Femoral neck BMD T-score | -1.17 (0.96) |

SD: standard deviation, BMD: bone mineral density.

**Table S2:** *Cis*-eQTL associations identified in the osteoclast-like cells for eBMD GWAS variants that were not co-localised

| **Variant** | **Location** | **EA** | **OA** | **EAF** | **β_GWAS_** | **Gene** | **Expression^a^** | **Distance to TSS** | ***P*_eQTL_** | **β_eQTL_** |
| --- | --- | --- | --- | --- | --- | --- | --- | --- | --- | --- |
| rs938295 | chr1:15760765 | T | C | 0.16 | 0.03 | *HSPB7* | 9.44 ± 8.68 | -258,830 | 2.72E-05 | 0.71 |
| rs79364962 | chr1:22205049 | A | G | 0.07 | -0.07 | *NBPF3* | 0.4 ± 0.29 | 764,921 | 3.05E-05 | 0.98 |
| rs7546500 | chr1:26163142 | G | T | 0.32 | 0.02 | *ZNF593* | 7.26 ± 1.7 | -6,729 | 2.16E-07 | -0.63 |
| rs4589135 | chr1:26715223 | C | T | 0.42 | -0.02 | *PIGV* | 4.22 ± 0.57 | -72,249 | 3.40E-05 | -0.45 |
| rs12031054 | chr1:66641142 | A | G | 0.27 | -0.02 | *TCTEX1D1* | 0.86 ± 0.88 | -111,317 | 1.34E-17 | 1.03 |
| rs167365 | chr1:88715162 | C | G | 0.37 | 0.02 | *KYAT3* | 12.72 ± 2.15 | -277,792 | 4.44E-08 | -0.63 |
| rs3790608 | chr1:112512401 | A | G | 0.13 | 0.04 | *ST7L* | 1.35 ± 0.36 | -108,425 | 9.94E-07 | 0.85 |
| rs56240884 | chr2:28717288 | T | C | 0.33 | 0.02 | *TRMT61B* | 4.67 ± 0.77 | -153,014 | 6.49E-10 | 0.70 |
| rs75475627 | chr2:54560455 | G | C | 0.08 | -0.09 | *SPTBN1* | 1.54 ± 1.03 | 104,170 | 9.38E-06 | 0.94 |
| rs2971879 | chr2:54661396 | T | C | 0.32 | -0.05 | *SPTBN1* | 1.54 ± 1.03 | 205,111 | 1.51E-07 | 0.61 |
| rs4470366 | chr2:71307487 | T | A | 0.38 | -0.01 | *DYSF* | 4.95 ± 3.71 | -146,235 | 1.75E-09 | 0.73 |
| rs62179714 | chr2:190702815 | C | G | 0.26 | -0.01 | *HIBCH* | 2.35 ± 0.61 | 358,621 | 1.95E-04 | -0.47 |
| rs11677953 | chr2:218256940 | A | G | 0.41 | 0.01 | *RP11-378A13.1* | 1.69 ± 0.48 | 1,621 | 1.13E-18 | -0.90 |
| rs11677953 | chr2:218256940 | A | G | 0.41 | 0.01 | *TMBIM1* | 108.46 ± 20.8 | -35,647 | 1.91E-04 | -0.42 |
| rs2432236 | chr5:72952892 | G | A | 0.45 | 0.01 | *FCHO2* | 31.54 ± 8.43 | -3,089 | 9.88E-15 | 0.78 |
| rs2432236 | chr5:72952892 | G | A | 0.45 | 0.01 | *CTD-2376I4.2* | 4.24 ± 2.39 | -2,808 | 1.88E-10 | 0.68 |
| rs2432236 | chr5:72952892 | G | A | 0.45 | 0.01 | *CTD-2376I4.1* | 1.06 ± 0.71 | -1,383 | 1.76E-06 | 0.52 |
| rs42916 | chr5:128224967 | C | A | 0.26 | -0.02 | *LINC01184* | 1.51 ± 0.3 | 141,794 | 1.30E-05 | -0.53 |
| rs42916 | chr5:128224967 | C | A | 0.26 | -0.02 | *SLC12A2* | 0.77 ± 0.21 | 141,201 | 3.25E-05 | -0.52 |
| rs10057855 | chr5:128340673 | A | G | 0.1 | -0.01 | *FBN2* | 0.18 ± 0.34 | -318,514 | 2.17E-08 | 1.02 |
| rs7747253 | chr6:29952002 | C | A | 0.44 | 0.02 | *PPP1R11* | 0.12 ± 0.23 | -114,707 | 7.78E-05 | 0.37 |
| rs2406255 | chr7:100456067 | C | T | 0.19 | -0.01 | *PILRB* | 0.23 ± 0.3 | 103,891 | 8.27E-10 | 0.77 |
| rs785836 | chr9:248428 | T | C | 0.31 | 0.01 | *CBWD1* | 0.4 ± 0.11 | 69,280 | 1.83E-06 | 0.58 |
| rs11814082 | chr10:30882400 | C | T | 0.21 | 0.01 | *DDX10P1* | 0.3 ± 0.17 | -38,836 | 1.22E-09 | 0.83 |
| rs11814082 | chr10:30882400 | C | T | 0.21 | 0.01 | *ZNF438* | 14.71 ± 2.06 | -149,538 | 1.68E-04 | -0.51 |
| rs11238526 | chr10:43379193 | G | A | 0.09 | 0.02 | *RP11-517P14.2* | 1.69 ± 0.64 | -41,545 | 1.43E-04 | 0.77 |
| rs12776318 | chr10:100108920 | G | T | 0.37 | 0.01 | *CWF19L1* | 5.01 ± 1.04 | -158,761 | 8.56E-09 | 0.61 |
| rs11191614 | chr10:103217846 | T | C | 0.16 | -0.01 | *BORCS7* | 5.75 ± 1.25 | 363,623 | 1.78E-08 | -0.88 |
| rs12254582 | chr10:129481527 | G | A | 0.29 | -0.02 | *MGMT* | 3.56 ± 0.88 | 14,343 | 2.62E-10 | -0.71 |
| rs174534 | chr11:61781986 | G | A | 0.36 | 0.02 | *FADS2* | 21.1 ± 6.96 | -10,994 | 4.14E-05 | 0.48 |
| rs17773169 | chr11:62551058 | A | C | 0.4 | -0.01 | *EML3* | 9.08 ± 2.43 | -61,708 | 2.71E-08 | 0.48 |
| rs12855887 | chr13:42985632 | G | A | 0.47 | 0.01 | *DNAJC15* | 3.9 ± 0.88 | -37,571 | 1.20E-06 | 0.56 |
| rs2153672 | chr13:99648688 | T | C | 0.08 | 0.02 | *CLYBL* | 2.44 ± 0.75 | 42,019 | 6.86E-05 | -0.74 |
| rs3759549 | chr14:34981088 | T | A | 0.5 | 0.02 | *SRP54* | 20.26 ± 3.2 | -869 | 4.54E-06 | -0.47 |
| rs2899472 | chr15:51223858 | A | C | 0.27 | 0.03 | *GLDN* | 0.05 ± 0.07 | -117,771 | 1.14E-07 | 0.64 |
| rs2899472 | chr15:51223858 | A | C | 0.27 | 0.03 | *CYP19A1* | 5.61 ± 5.31 | -114,753 | 3.13E-06 | 0.59 |
| rs12325187 | chr16:3314997 | G | C | 0.24 | 0.01 | *ZNF200* | 1.63 ± 0.37 | 78,775 | 6.25E-05 | 0.54 |
| rs12325187 | chr16:3314997 | G | C | 0.24 | 0.01 | *ZNF174* | 2.44 ± 0.31 | -86,238 | 1.81E-04 | 0.50 |
| rs3901638 | chr16:4310283 | G | A | 0.31 | -0.01 | *NLRC3* | 1.59 ± 2.03 | 732,882 | 1.02E-04 | 0.46 |
| rs1703492 | chr16:12043097 | A | C | 0.33 | -0.01 | *RP11-166B2.1* | 0.16 ± 0.16 | 66,453 | 1.46E-16 | 0.94 |
| rs17249128 | chr16:51034374 | G | A | 0.49 | 0.01 | *HEATR3* | 11.91 ± 1.95 | 968,433 | 5.95E-05 | -0.45 |
| rs57696383 | chr16:89506257 | G | A | 0.42 | -0.01 | *RP11-104N10.2* | 0.42 ± 0.37 | -10,540 | 2.69E-06 | 0.42 |
| rs11658168 | chr17:7502815 | A | G | 0.31 | -0.01 | *CHRNB1* | 1.35 ± 0.26 | 57,754 | 1.07E-06 | 0.61 |
| rs12452440 | chr17:82569480 | T | C | 0.38 | -0.02 | *FOXK2* | 8.64 ± 1.1 | 49,767 | 1.51E-06 | -0.53 |
| rs856999 | chr20:10574758 | G | C | 0.35 | 0.01 | *SLX4IP* | 1.1 ± 0.3 | 139,455 | 3.27E-05 | 0.43 |
| rs6059412 | chr20:33693023 | T | G | 0.32 | 0.02 | *SNTA1* | 1.59 ± 0.57 | 249,130 | 9.86E-05 | 0.48 |
| rs9975345 | chr21:46423090 | T | C | 0.47 | 0.01 | *YBEY* | 4.78 ± 1.58 | 136,753 | 3.47E-14 | -0.71 |
| rs139497 | chr22:41244094 | C | T | 0.31 | -0.02 | *RP4-756G23.5* | 0.53 ± 0.42 | 26,466 | 5.18E-07 | 0.59 |

EA: effect allele, OA: other allele, EAF: effect allele frequency (derived from the osteoclast eQTL cohort), eBMD: estimated BMD, TSS: transcription start site, variant locations derived from dbSNP build 150 (GRCh38/hg38), β_GWAS_ values are relevant to the effect allele and were obtained from Morris et al. (1), β_eQTL_ values are given as the normalised effect size on gene expression for the effect allele. eQTL associations are significant using a multiple testing corrected FDR of 5%.

^a^Expression levels are stated as mean reads per kilobase million (RPKM) ± standard deviation.

**Table S3:** Significant associations identified in the SMR analysis of the eBMD GWAS and osteoclast eQTL datasets

| **Chr** | **Gene** | ***Cis*-eQTL** | ***P*_GWAS_** | ***P*_eQTL_** | ***P*_SMR_** | **β_SMR_** | ***P*_HEIDI_** |
| --- | --- | --- | --- | --- | --- | --- | --- |
| 1 | *LINC00339* | rs2501289 | 1.80E-09 | 3.35E-22 | 6.59E-07 | -0.01 | 1.60E-09 |
| 1 | *TCTEX1D1* | rs657808 | 3.40E-13 | 8.04E-23 | 2.65E-09 | 0.01 | 1.36E-15 |
| 1 | *CRYZ* | rs11485298 | 1.10E-06 | 2.20E-40 | 2.52E-06 | -0.01 | 1.78E-09 |
| 1 | *KYAT3* | rs28633936 | 7.10E-14 | 3.56E-23 | 1.57E-09 | 0.01 | 9.15E-17 |
| 1 | *ST7L* | rs10776756 | 5.70E-15 | 1.38E-36 | 1.32E-11 | 0.01 | 1.07E-27 |
| 1 | *RRP15* | rs10863387 | 5.90E-09 | 2.14E-16 | 5.12E-06 | 0.01 | 2.95E-04 |
| 2 | *TRMT61B* | rs6709674 | 6.40E-17 | 5.92E-17 | 1.03E-08 | -0.02 | 1.24E-17 |
| 2 | *SPTBN1* | rs354214 | 8.30E-111 | 3.23E-09 | 1.14E-08 | 0.07 | 0 |
| 2 | *TEX41* | rs12467931 | 1.10E-14 | 1.13E-08 | 5.27E-06 | -0.02 | 4.56E-15 |
| 2 | *RP11-378A13.1* | rs56344368 | 1.90E-09 | 4.63E-22 | 2.66E-07 | 0.01 | 1.98E-08 |
| 3 | *NCKIPSD* | rs1352420 | 5.60E-20 | 1.40E-14 | 1.10E-08 | -0.02 | 5.20E-09 |
| 3 | *WDR6* | rs9311434 | 1.60E-20 | 2.27E-22 | 1.30E-10 | 0.02 | 1.07E-15 |
| 4 | *CPE* | rs1370683 | 7.90E-11 | 2.38E-10 | 4.32E-06 | 0.02 | 4.13E-07 |
| 5 | *CTD-2376I4.2* | rs703869 | 1.40E-09 | 3.67E-11 | 6.69E-06 | -0.02 | 3.17E-09 |
| 5 | *FCHO2* | rs703869 | 1.40E-09 | 2.45E-15 | 1.20E-06 | -0.01 | 3.34E-09 |
| 5 | *LINC01184* | rs2250127 | 1.40E-05 | 1.81E-18 | 2.91E-05 | 0.01 | 1.39E-06 |
| 6 | *BTN3A2* | rs9393703 | 1.20E-10 | 5.21E-19 | 2.97E-07 | -0.01 | 8.80E-03 |
| 6 | *VNN2* | rs6569844 | 1.40E-11 | 1.69E-18 | 1.06E-07 | 0.01 | 2.03E-17 |
| **6** | ***TULP4*** | **rs341106** | **9.00E-09** | **1.70E-08** | **4.43E-05** | **0.02** | **7.26E-01** |
| 7 | *BRI3* | rs112758337 | 2.50E-06 | 7.39E-14 | 4.35E-05 | 0.01 | 2.53E-06 |
| 7 | *PILRB* | rs11765869 | 1.50E-10 | 2.08E-10 | 7.79E-06 | -0.02 | 5.27E-05 |
| 8 | *SGK223* (*PRAG1*) | rs4240617 | 2.10E-12 | 1.38E-15 | 2.03E-07 | -0.02 | 2.80E-12 |
| 8 | *AF131215.2* | rs13276836 | 5.80E-72 | 9.16E-11 | 1.18E-09 | 0.05 | 0 |
| 9 | *CBWD1* | rs644383 | 1.30E-06 | 1.59E-16 | 3.06E-05 | 0.01 | 8.65E-07 |
| 10 | *DDX10P1* | rs3006594 | 3.70E-13 | 3.75E-12 | 7.37E-07 | 0.02 | 6.02E-04 |
| 10 | *STOX1* | rs12268540 | 4.30E-07 | 2.82E-24 | 8.97E-06 | -0.01 | 2.36E-03 |
| 10 | *BORCS7* | rs11191421 | 7.50E-07 | 4.75E-19 | 1.14E-05 | 0.01 | 4.00E-02 |
| 10 | *METTL10* | rs2303611 | 1.10E-11 | 6.83E-12 | 2.79E-06 | 0.02 | 1.33E-04 |
| 10 | *MGMT* | rs1008982 | 1.00E-07 | 1.89E-17 | 1.20E-05 | 0.01 | 1.02E-10 |
| 10 | *DPYSL4* | rs7914962 | 9.10E-08 | 2.95E-11 | 2.27E-05 | -0.01 | 1.43E-03 |
| 11 | *ARL14EP* | rs522089 | 4.70E-07 | 1.13E-17 | 1.73E-05 | 0.01 | 6.88E-06 |
| 11 | *EML3* | rs1801144 | 5.90E-10 | 6.00E-09 | 2.92E-05 | -0.02 | 2.01E-03 |
| 11 | *GAL* | rs1546309 | 1.00E-14 | 2.61E-08 | 1.05E-05 | 0.02 | 5.20E-06 |
| 11 | *RP11-757G1.6* | rs583182 | 1.20E-11 | 9.48E-14 | 8.03E-07 | -0.01 | 3.38E-10 |
| 11 | *MRPL21* | rs646586 | 3.90E-12 | 2.37E-20 | 5.46E-08 | -0.01 | 4.20E-12 |
| 11 | *AP002954.4* | rs4938523 | 1.70E-10 | 4.57E-17 | 7.70E-07 | -0.01 | 1.43E-08 |
| 12 | *RP11-218M22.1* | rs55800929 | 1.20E-06 | 6.37E-22 | 2.08E-05 | -0.01 | 9.05E-06 |
| 13 | *DNAJC15* | rs2281780 | 4.40E-09 | 2.28E-36 | 8.06E-08 | 0.01 | 2.36E-06 |
| 14 | *EIF2B2* | rs175438 | 1.60E-18 | 1.32E-11 | 7.85E-08 | 0.02 | 8.80E-15 |
| 14 | *APOPT1* | rs28513222 | 5.60E-53 | 3.63E-09 | 4.17E-08 | -0.05 | 2.50E-16 |
| 14 | *RP11-73M18.7* | rs861544 | 3.20E-14 | 9.21E-09 | 6.39E-06 | 0.02 | 6.64E-12 |
| 15 | *SPPL2A* | rs151067682 | 1.10E-09 | 2.95E-08 | 4.24E-05 | -0.02 | 7.51E-05 |
| 15 | *CYP19A1* | rs11856927 | 1.10E-37 | 4.95E-12 | 1.29E-09 | -0.03 | 1.72E-23 |
| 15 | *GLDN* | rs8039089 | 1.10E-56 | 2.75E-16 | 4.51E-13 | 0.04 | 0 |
| 15 | *GDPGP1* | rs1044246 | 5.10E-12 | 2.29E-09 | 1.48E-05 | -0.02 | 4.29E-05 |
| 15 | *IQGAP1* | rs2074585 | 6.60E-31 | 1.21E-11 | 8.03E-09 | -0.03 | 1.29E-09 |
| 16 | *TMEM8A* | rs8064205 | 6.70E-24 | 1.34E-10 | 7.95E-08 | -0.02 | 1.04E-19 |
| 17 | *WDR81* | rs2287322 | 9.70E-07 | 1.78E-31 | 7.22E-06 | 0.01 | 2.94E-05 |
| 17 | *SRR* | rs3744270 | 1.10E-07 | 6.21E-25 | 5.65E-06 | -0.01 | 5.85E-10 |
| 17 | *FLCN* | rs1708623 | 1.20E-13 | 7.18E-11 | 1.58E-06 | 0.02 | 1.17E-06 |
| 17 | *SPATA20* | rs9890200 | 2.90E-08 | 5.80E-37 | 1.28E-06 | 0.01 | 8.73E-05 |
| 19 | *PRDX2* | rs897804 | 2.10E-09 | 8.22E-16 | 1.27E-06 | 0.01 | 2.54E-06 |
| 19 | *MBOAT7* | rs36655 | 6.00E-06 | 1.48E-30 | 3.31E-05 | 0.01 | 1.50E-04 |

A Bonferroni multiple-testing corrected threshold of *P*_SMR_ *<4.7×10^-5^* was used to identify significant associations, bold text highlights *P*_HEIDI_ *>0.05*.

**
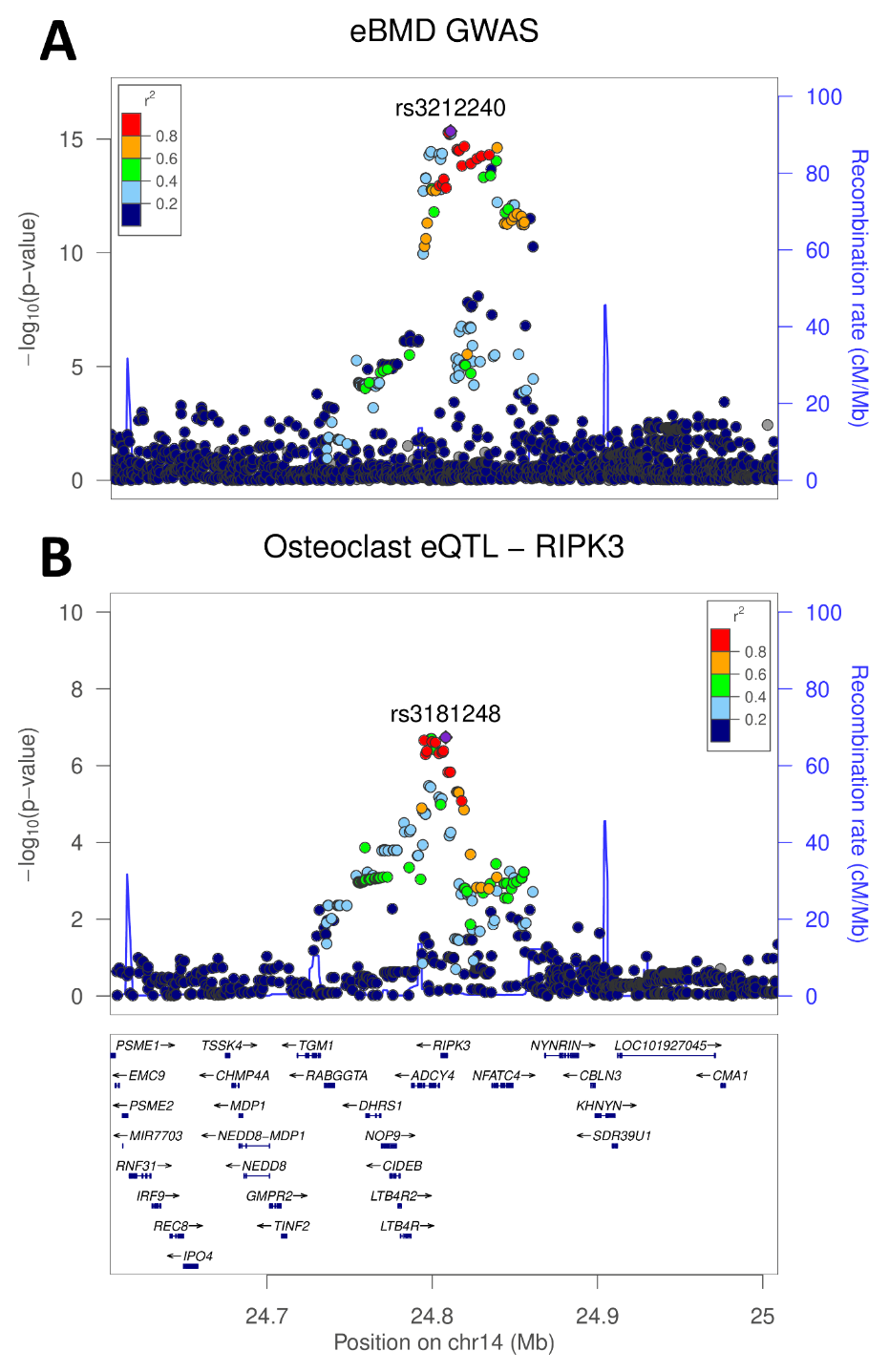
**

**Fig. S1.** Association plots generated using (**A**) eBMD GWAS association results from Morris et al. (1) and (**B**) osteoclast eQTL association results for the *RIPK3* gene. Analysis of the datasets using the coloc software (2) generated strong evidence (89.8% posterior probability) for co-localisation of the eBMD GWAS and osteoclast eQTL association signals. Genetic variants within 200 kb of the *RIPK3* gene are depicted (x axis) along with their association *P* value (–log10). The variants are colour coded according to their LD (r^2^) with the lead variant (1000GP Nov 2014 EUR population). The recombination rate (blue line) and position of genes, their exons and direction of transcription is also indicated (3).

**
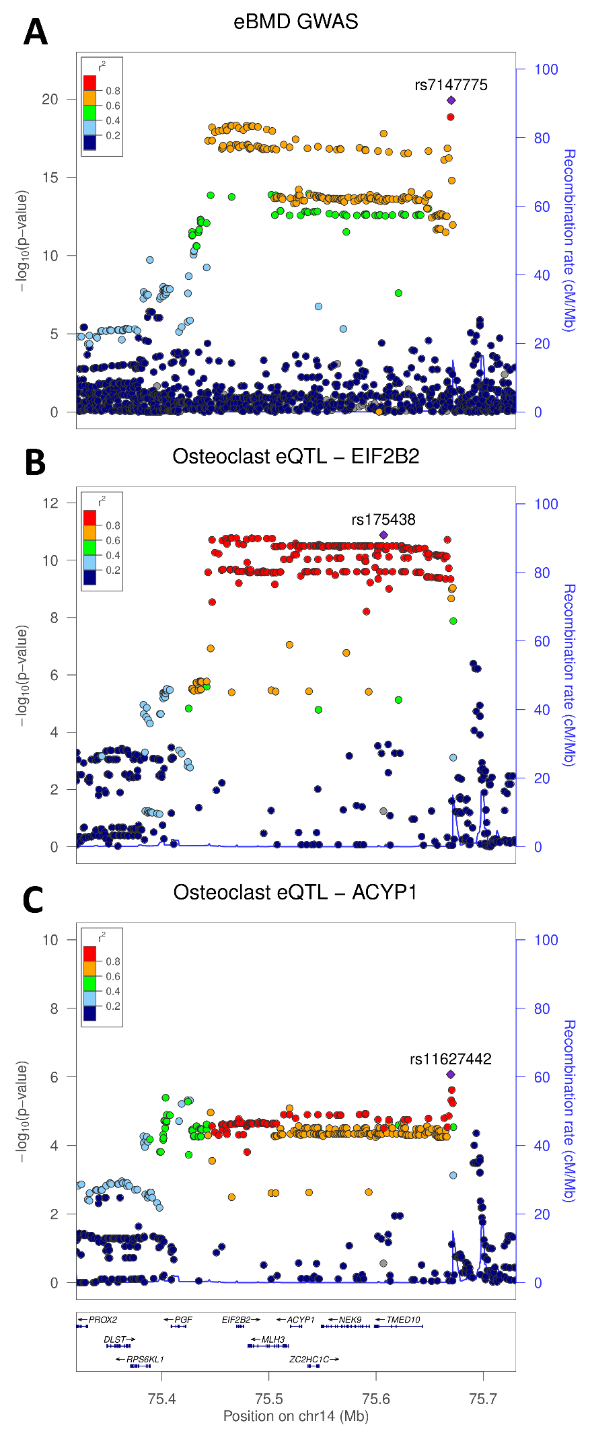
**

**Fig. S2.** Association plots generated using (**A**) eBMD GWAS association results from Morris et al. (1) and osteoclast eQTL association results for the (**B**) *EIF2B2* and (**C**) *ACYP1* genes. Co-localisation of eBMD GWAS and osteoclast eQTL association signals was identified for this locus using the coloc software (2) (65.9% and 83.4% posterior probability for the *EIF2B2* and *ACYP1* genes respectively). The top eBMD GWAS variant (rs7147775) is in moderate/strong LD with the top eQTL variants for the *EIF2B2* (rs175438, r^2^=0.73) and *ACYP1* genes (rs11627442, r^2^=0.81) in Europeans (4). Genetic variants within 200 kb of the *ACYP1* gene are depicted (x axis) along with their association *P* value (–log10).

**
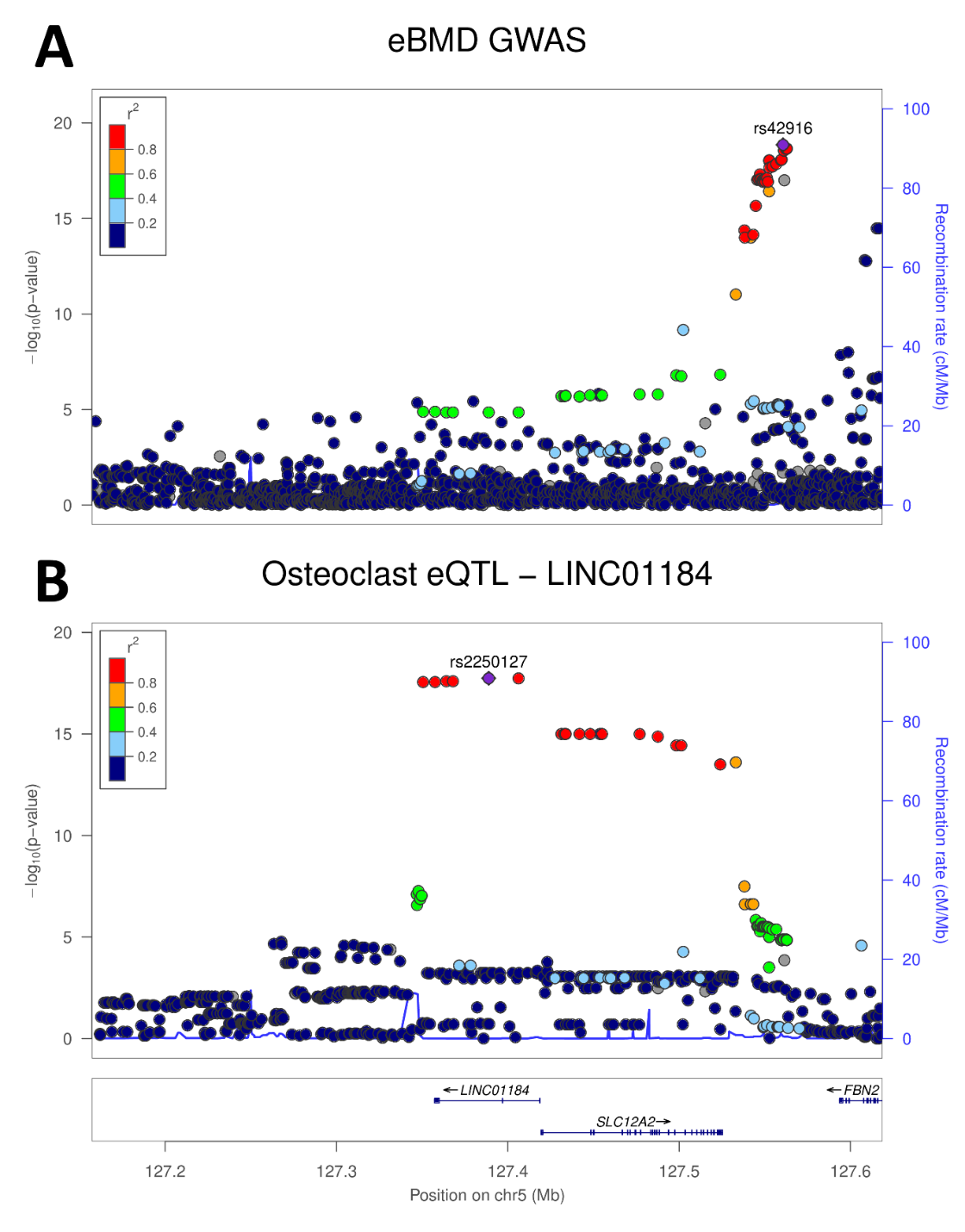
**

**Fig. S3.** An example of a genetic locus demonstrating independent eBMD GWAS and osteoclast eQTL association signals. Association plots generated using (**A**) eBMD GWAS association results from Morris et al. (1) and (**B**) osteoclast eQTL association results for the *LINC01184* gene. Co-localisation of eBMD GWAS and osteoclast eQTL association signals was deemed highly unlikely for this locus using the coloc software (2) (<0.01% posterior probability). Note the presence of independent association signals in the two datasets, led by rs42916 (GWAS) and rs2250127 (eQTL). Genetic variants within 200 kb of the *LINC01184* gene are depicted (x axis) along with their association *P* value (–log10).

**
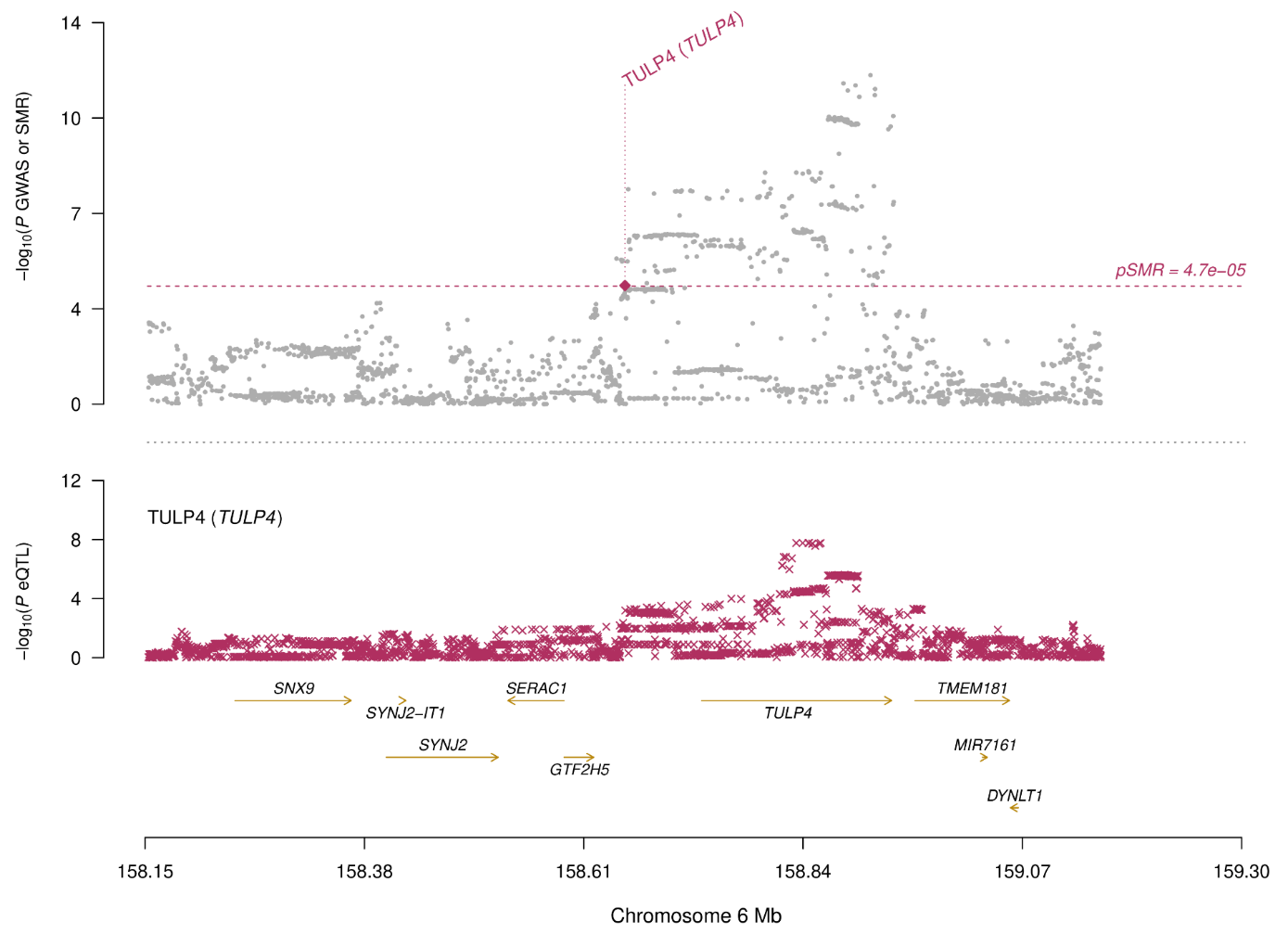
**

**Fig. S4.** SMR locus plot for the *TULP4* gene region demonstrating potential pleiotrophic effects on *TULP4* gene expression and eBMD. The eBMD GWAS *P* values (1) are plotted in the top frame, with the osteoclast eQTL *P* values for the *TULP4* gene presented in the middle frame and gene locations presented in the lower frame. The diamond represents the *P* value for the *TULP4* gene from the SMR test (*P*_SMR_ *= 4.43×10^-5^*, Additional file 1: Table S3), with the significance threshold indicated.

**

Fig. S5.** Trabecular (**A**) and cortical (**B**) micro-CT analysis results from 15 week-old male WT and *Ripk3^-/-^* mice (mean + standard deviation). N=5 for each group, WT: wildtype, BMD: bone mineral density, Tb.N: trabecular number, Tb.Th: trabecular thickness, Tb.Sp: trabecular separation, TMD: tissue mineral density, Ct.Th: cortical thickness.

**
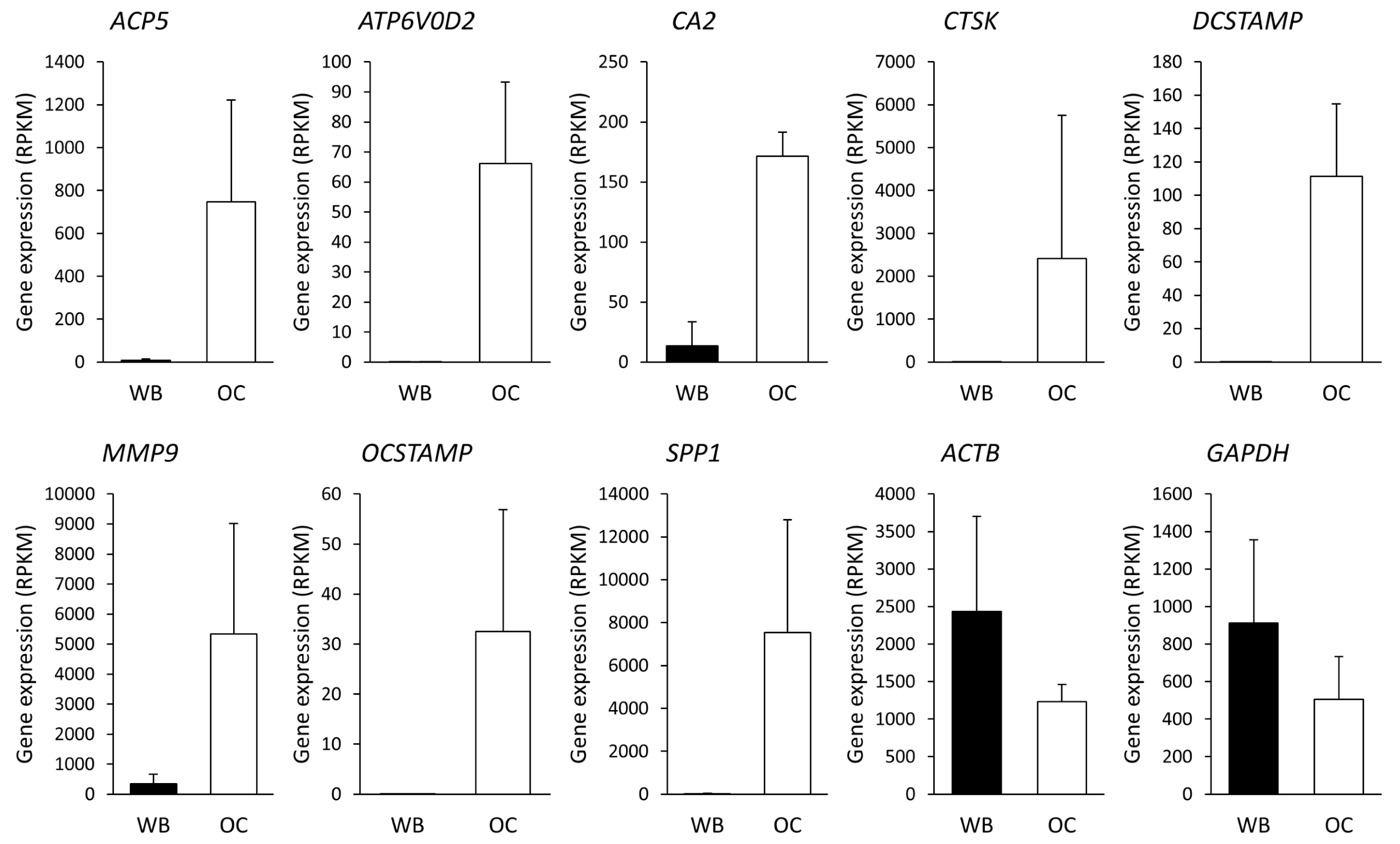
**

**Fig. S6.** Series of bar charts comparing the expression levels (mean + standard deviation) of numerous osteoclast marker genes in the osteoclast-like cells relative to that in the GTEx whole-blood dataset (n=393), with the commonly used housekeeping genes *ACTB* and *GAPDH* included for comparison. WB: whole-blood, OC: osteoclast.

**References**

1. Morris JA, Kemp JP, Youlten SE, Laurent L, Logan JG, Chai RC, et al. An atlas of genetic influences on osteoporosis in humans and mice. Nature genetics. 2019;51(2):258-66.

2. Giambartolomei C, Vukcevic D, Schadt EE, Franke L, Hingorani AD, Wallace C, et al. Bayesian test for colocalisation between pairs of genetic association studies using summary statistics. PLoS genetics. 2014;10(5):e1004383.

3. Pruim RJ, Welch RP, Sanna S, Teslovich TM, Chines PS, Gliedt TP, et al. LocusZoom: regional visualization of genome-wide association scan results. Bioinformatics (Oxford, England). 2010;26(18):2336-7.

4. Machiela MJ, Chanock SJ. LDlink: a web-based application for exploring population-specific haplotype structure and linking correlated alleles of possible functional variants. Bioinformatics (Oxford, England). 2015;31(21):3555-7.
