# Supplementary material for: Characterisation of genetic regulatory effects for osteoporosis risk variants in human osteoclasts
Source: Genome Biol. 2020 Mar 26;21:80. doi: 10.1186/s13059-020-01997-2 (PMC7098081; doi:10.1186/s13059-020-01997-2)
Supplement: Supplementary file 2 — Additional file 2. Review history. [file 13059_2020_1997_MOESM2_ESM.docx]

Review History

**First round of review**

**Reviewer 1**

**Comments to author:**

The article by Mullin et al. uses a unique osteoclast eQTL dataset to identify genes with eQTL that colocalize with eBMD associations identified by GWAS. The article is well-written and represents an important contribution to our understanding of the genetics of bone mass. I do have a few critiques for the authors to consider.

Critiques:

1.     In the first section of the results, the authors identify eQTL associations for the 929 lead eBMD SNPs identified by Morris et al. The authors then conduct a colocalization analysis. The value of the first set of results is unclear. Many of the association represent cis eQTL that are completely independent of the eBMD locus. I would recommend removing these data and simply reporting the colocalization analysis results for those genes with significantly colocalizing eQTL (also include these results in Table 1 instead of all the cis eQTL results). These are really the only results that matter and may be misleading to a reader without expertise in these kinds of analyses.
2.     There are a number of new colocalization methods (coloc2, enloc) that are designed to address the high levels of allelic heterogeneity present in data such as those from the Morris et al. study. The authors should consider using these new tools. The implementation of one of these tools would be very straightforward.
3.     I find it interesting that of the 929 associations interrogated only 19 (2.0%) were found to have at least one gene with a colocalizing eQTL in osteoclasts.  Given that osteoclasts are (obviously) a major driver of bone mass, I find this to be quite low. The authors should discuss why this result is so low. Is it statistical power? Is the contribution of osteoclasts to cross-sectional BMD (and eBMD; where one might think the trait is primarily a function of bone accrual) low? If the trait investigated by GWAS was bone loss would the results be different?
4.     The direction of effect of RIPK3 given the GWAS and eQTL data should be stated in the results section to clearly articulate the hypothesis being tested. It is mentioned in the discussion, but should be part of the results.
5.     Given the lack of a major cortical or trabecular bone phenotype in RIPK3 mice, the authors should discuss how the observed phenotype could relate to BMD (or eBMD) in humans. As presented these data do not necessarily reflect a role for RIPK3 in the regulation of BMD (or eBMD). The increased OC numbers is supportive, but a stronger case would have been made if a cortical or trabecular phenotype was observed.

**Reviewer 2**

**Comments to author:**

Mullin et al report a largely computational study designed to ask to what extent osteoclast eQTL overlap with GWAS peaks for bone mineral density.  Of 69 cases of overlap, they find strong evidence for colocalization at 19 loci, and provide additional evidence for enrichment of functional signals involved in bone development at various significance levels.  Histological analysis of one implicated gene, RIPK3, suggests a likely mechanism for its association with osteoporosis.  

The paper is well written and uses state-of-the-art software to ask the various questions.  The logical flow is coherent, and the message strikes me as well supported and of general interest to the readership of Genome Biology.  I have just a couple of points of clarification to recommend, and one suggestion for further analysis that could provide additional insight.

While the sections on coloc and SMR in the Methods are clear enough, I would recommend that some additional description and interpretation be provided in the Results.  In my experience, non-experts tend to assume there is a single eQTL per gene and hence that where there is a GWAS and an eQTL signal at the same locus, they must be the same.  A few sentences, and perhaps a figure illustrating one example of H4 > 0.8 and one H4 < 0.2 would help.  Also note that the significance criteria for H4 posterior probability >0.5 that the authors use is just a guideline; with a smallish eQTL dataset, power is relatively low: the fact that 2/3 of the cases do not show significant coloc results does not necessarily imply independent signals.  SMR is even more susceptible to small sample (modest p-value) biases, and readers should be given some guidance in interpreting the very high percentage of significant HEIDI scores, presumably including several of those with significant coloc results.

My analytical suggestion is to explore further the concordance of sign effects of eQTL and GWAS betas, along the lines of the coherence/incoherence analysis of Marigorta et al (Nature Genetics 2017, PMID PMID: 28805827).  They asked whether the sign of association of the eQTL and case (IBD) status is the same as the observed differential expression.  For example, if the risk allele increases expression the expectation is that cases will have higher expression, but quite often that is not the case, which they argue indicates that the GWAS effect is due to inability to induce (or reduce) the expression sufficiently to provide protection.  I think that a similar analysis could be performed here by looking at the direction of association of the risk allele with higher or lower bone density, and the direction of association of gene expression with BMD in their cohort, then matched to the eQTL.

**Authors Response**

**Point-by-point responses to the reviewers’ comments:**

**Reviewer 1**

1) In the first section of the results, the authors identify eQTL associations for the 929 lead eBMD SNPs identified by Morris et al. The authors then conduct a colocalization analysis. The value of the first set of results is unclear. Many of the association represent cis eQTL that are completely independent of the eBMD locus. I would recommend removing these data and simply reporting the colocalization analysis results for those genes with significantly colocalizing eQTL (also include these results in Table 1 instead of all the cis eQTL results). These are really the only results that matter and may be misleading to a reader without expertise in these kinds of analyses.

*Response: We have edited Table 1 so that it only contains the results for the genetic loci that demonstrated co-localisation of eBMD GWAS and eQTL association signals. The results for the remaining loci have been retained for completeness, but moved to Supplementary Table S2.*

2) There are a number of new colocalization methods (coloc2, enloc) that are designed to address the high levels of allelic heterogeneity present in data such as those from the Morris et al. study. The authors should consider using these new tools. The implementation of one of these tools would be very straightforward.

*Response: We have re-run the co-localisation analysis using the coloc2 software and updated the Abstract (page 2, line 12), Methods (page 19, lines 20-23), Results (page 7, line 20 – page 8, line 3) and Discussion (page 12, line 18) sections of the manuscript accordingly.*

3) I find it interesting that of the 929 associations interrogated only 19 (2.0%) were found to have at least one gene with a colocalizing eQTL in osteoclasts. Given that osteoclasts are (obviously) a major driver of bone mass, I find this to be quite low. The authors should discuss why this result is so low. Is it statistical power? Is the contribution of osteoclasts to cross-sectional BMD (and eBMD; where one might think the trait is primarily a function of bone accrual) low? If the trait investigated by GWAS was bone loss would the results be different?

*Response: The relatively low proportion of co-localised loci identified in the study is potentially due to a combination of factors, including the statistical power of the osteoclast eQTL cohort, the relative contribution of osteoclasts to the eBMD trait and the smaller number of expressed genes in the differentiated osteoclast-like cell cultures when compared to complex tissues containing a mixture of cell types (eg. GTEx tissues). We have added an interpretation of these factors to the Discussion (page 14, line 22 – page 15, line 3).*

4) The direction of effect of RIPK3 given the GWAS and eQTL data should be stated in the results section to clearly articulate the hypothesis being tested. It is mentioned in the discussion, but should be part of the results.

*Response: We have added this information to the Results section (page 9, lines 22- 24).*

5) Given the lack of a major cortical or trabecular bone phenotype in RIPK3 mice, the authors should discuss how the observed phenotype could relate to BMD (or eBMD) in humans. As presented these data do not necessarily reflect a role for RIPK3 in the regulation of BMD (or eBMD). The increased OC numbers is supportive, but a stronger case would have been made if a cortical or trabecular phenotype was observed.

*Response: We have now addressed this issue in the Discussion (page 11, lines 18- 24).*

**Reviewer 2**

1) While the sections on coloc and SMR in the Methods are clear enough, I would recommend that some additional description and interpretation be provided in the Results. In my experience, non-experts tend to assume there is a single eQTL per gene and hence that where there is a GWAS and an eQTL signal at the same locus, they must be the same. A few sentences, and perhaps a figure illustrating one example of H4 > 0.8 and one H4 < 0.2 would help. Also note that the significance criteria for H4 posterior probability >0.5 that the authors use is just a guideline; with a smallish eQTL dataset, power is relatively low: the fact that 2/3 of the cases do not show significant coloc results does not necessarily imply independent signals. SMR is even more susceptible to small sample (modest p-value) biases, and readers should be given some guidance in interpreting the very high percentage of significant HEIDI scores, presumably including several of those with significant coloc results.

*Response: We have added information regarding co-localised vs independent association signals to the Results section (page 7, lines 8-13), and an example of a locus displaying independent signals has been added to the Supplementary Material (Supplementary Figure S3). We have also added some comments regarding use of the co-localisation posterior probability threshold and the effect of the osteoclast eQTL sample size on the co-localisation and SMR analyses to the Discussion (page 15, lines 3-8).*

2) My analytical suggestion is to explore further the concordance of sign effects of eQTL and GWAS betas, along the lines of the coherence/incoherence analysis of Marigorta et al (Nature Genetics 2017, PMID PMID: 28805827). They asked whether the sign of association of the eQTL and case (IBD) status is the same as the observed differential expression. For example, if the risk allele increases expression the expectation is that cases will have higher expression, but quite often that is not the case, which they argue indicates that the GWAS effect is due to inability to induce (or reduce) the expression sufficiently to provide protection. I think that a similar analysis could be performed here by looking at the direction of association of the risk allele with higher or lower bone density, and the direction of association of gene expression with BMD in their cohort, then matched to the eQTL.

*Response: We read the interesting manuscript by Marigorta et al. and decided to attempt a similar analysis using our datasets. We performed a differential gene expression analysis on the osteoclast RNA-seq data using the edgeR package, comparing gene expression in the 20 highest vs 20 lowest femoral neck DXA BMD individuals in our osteoclast eQTL cohort. Unfortunately, none of the co-localised genes identified in our manuscript were found to be significantly differentially expressed between the two groups, likely due to the different phenotype used (ie. DXA BMD vs eBMD) and insufficient statistical power. Thank you for this interesting suggestion; we will consider the technique, where suitable, in future studies.*

**Second round of review**

**Reviewer 1**

The authors have satisfactorily responded to my prior comments and suggestions.

**Reviewer 2**

A pity that the coherence analysis did not work out, but thanks for trying.  The low proportion of colocalized signals is also a bit of a concern, but certainly not specific to this study, it seems to be a general feature of combined eQTL/GWAS analysis.  The analyses reported here are nevertheless a useful addition to the literature, and they seem to have been performed well.  I am well satisfied with the remaining responses
